# Hepatocyte mitochondrial NAD^+^ content is limiting for liver regeneration

**DOI:** 10.1038/s42255-025-01408-5

**Published:** 2025-11-20

**Authors:** Sarmistha Mukherjee, Ricardo A. Velázquez Aponte, Caroline E. Perry, Won Dong Lee, Kevin A. Janssen, Marc Niere, Gabriel K. Adzika, Mu-Jie Lu, Hsin-Ru Chan, Xiangyu Zou, Beishan Chen, Nicole Bye, Teresa Xiao, Jin-Seon Yook, Oniel Salik, David W. Frederick, Ryan B. Gaspar, Khanh V. Doan, James G. Davis, Joshua D. Rabinowitz, Douglas C. Wallace, Nathaniel W. Snyder, Shingo Kajimura, Xiaolu A. Cambronne, Mathias Ziegler, Joseph A. Baur

**Affiliations:** 1 Department of Physiology and Institute for Diabetes, Obesity and Metabolism, Perelman School of Medicine, University of Pennsylvania, Philadelphia, PA USA; 2 Ludwig Institute for Cancer Research, Lewis–Sigler Institute for Integrative Genomics, Department of Chemistry, Princeton University, Princeton, NJ USA; 3 Department of Biochemistry, Yonsei University, Seoul, South Korea; 4 Department of Pediatrics, Division of Human Genetics, Perelman School of Medicine, University of Pennsylvania, Philadelphia, PA USA; 5 Department of Biomedicine, University of Bergen, Bergen, Norway; 6 Department of Molecular Biosciences, University of Texas at Austin, Austin, TX USA; 7 Division of Endocrinology, Diabetes and Metabolism, Beth Israel Deaconess Medical Center and Harvard Medical School, and Howard Hughes Medical Institute, Boston, MA USA; 8 Aging and Cardiovascular Discovery, Lewis Katz School of Medicine, Temple University, Philadelphia, PA USA; 9 Center for Mitochondrial and Epigenomic Medicine, Children’s Hospital of Philadelphia, Philadelphia, PA USA

**Keywords:** Energy metabolism, Metabolomics, Homeostasis

## Abstract

Nicotinamide adenine dinucleotide (NAD^+^) precursor supplementation shows metabolic and functional benefits in rodent models of disease and is being explored as potential therapeutic strategy in humans. However, the wide range of processes that involve NAD^+^ in every cell and subcellular compartment make it difficult to narrow down the mechanisms of action. Here we show that the rate of liver regeneration is closely associated with the concentration of NAD^+^ in hepatocyte mitochondria. We find that the mitochondrial NAD^+^ concentration in hepatocytes of male mice is determined by the expression of the transporter SLC25A51 (MCART1). The heterozygous loss of SLC25A51 modestly decreases mitochondrial NAD^+^ content in multiple tissues and impairs liver regeneration, whereas the hepatocyte-specific overexpression of SLC25A51 is sufficient to enhance liver regeneration comparably to the effect of systemic NAD^+^ precursor supplements. This benefit is observed even though NAD^+^ levels are increased only in mitochondria. Thus, the hepatocyte mitochondrial NAD^+^ pool is a key determinant of the rate of liver regeneration.

## Abstract (web summary)

Modulating mitochondrial NAD^+^ levels by changing the expression of the mitochondrial NAD^+^ transporter, SLC25A51, Mukherjee et al. demonstrate that mitochondrial, rather than cytosolic or nuclear, NAD^+^ levels are a key determinant of the rate of liver regeneration.

## Main

Nicotinamide adenine dinucleotide (NAD^+^) is a fundamental cofactor for reduction–oxidation reactions that is essential in all living cells^1^. It is the direct precursor to a phosphorylated form, NADP^+^, that serves as the cofactor in a distinct set of reactions, and it serves as a cosubstrate for several families of enzymes involved in cellular signalling. These include ADP-ribosyltransferases (ARTs), sirtuins (SIRT1–SIRT7) and glycohydrolases such as CD38 and sterile alpha and TIR motif containing 1 (SARM1), all of which break down the chemical backbone of NAD^+^, releasing nicotinamide. To maintain the pool of NAD^+^, most cells use the NAD^+^ salvage pathway, which converts nicotinamide to its mononucleotide (NMN) and then to NAD^+^ via the sequential action of nicotinamide phosphoribosyltransferase and NMN adenylyltransferases (NMNAT1–NMNAT3). In addition, the NAD^+^ pool is augmented by de novo synthesis from tryptophan and by the incorporation of nicotinic acid via the Preiss–Handler pathway^1^.

The balance between NAD^+^ synthesis and breakdown may be disrupted under stressful or pathological conditions, and it is hypothesized that the resultant loss of NAD^+^ and related molecules might contribute to further dysfunction^2^. Indeed, in many rodent models of disease, NAD^+^ levels are lower and supplying exogenous NAD^+^ precursors is therapeutic, even when it is not clear how they would affect the primary defect^3–5^. In human clinical trials, results have been mixed, often failing to recapitulate rodent outcomes^6^. However, promising early results have emerged for several indications, including mitochondrial myopathy^7^, ataxia telangiectasia^8^ and peripheral artery disease^9^. As the delivery is systemic in both rodents and humans, and NAD^+^ or its metabolites are involved in hundreds of reactions within each cell type, it is challenging to assign a mechanism of action for therapeutic effects in most cases. The problem is compounded by the fact that even within a given cell, NAD^+^ is compartmentalized, and there is little understanding of which pool is most relevant to outcomes or what governs the subcellular distribution of NAD^+^ (refs. ^1,10^). An exception is the recent discovery that the mitochondrial NAD^+^ pool in cultured cells is generated by import from the cytosol via SLC25A51^11–13^. This affords an opportunity to manipulate and study the mitochondrial NAD^+^ pool within specific cell types.

Here, we used gain and loss of function models to show that SLC25A51 expression determines mitochondrial NAD^+^ content in intact mouse liver. Supplementation with NAD^+^ precursors is well established to promote regeneration after partial hepatectomy (PHx)^14–16^, and we have previously shown that the effect can be mimicked by increasing NAD^+^ synthesis, specifically in hepatocytes^14^. We therefore used this system to test whether the mitochondrial NAD^+^ pool might account for the beneficial effect of NAD^+^ supplements on the regenerative capacity of the liver. We find that haploinsufficiency for SLC25A51 lowers mitochondrial NAD^+^ and impairs regeneration despite unchanged whole-tissue NAD^+^ levels and, conversely, that overexpression of SLC25A51 raises mitochondrial NAD^+^ content and improves regeneration comparably with the effect of systemic NAD^+^ precursor supplementation.

**Fig. 1:**
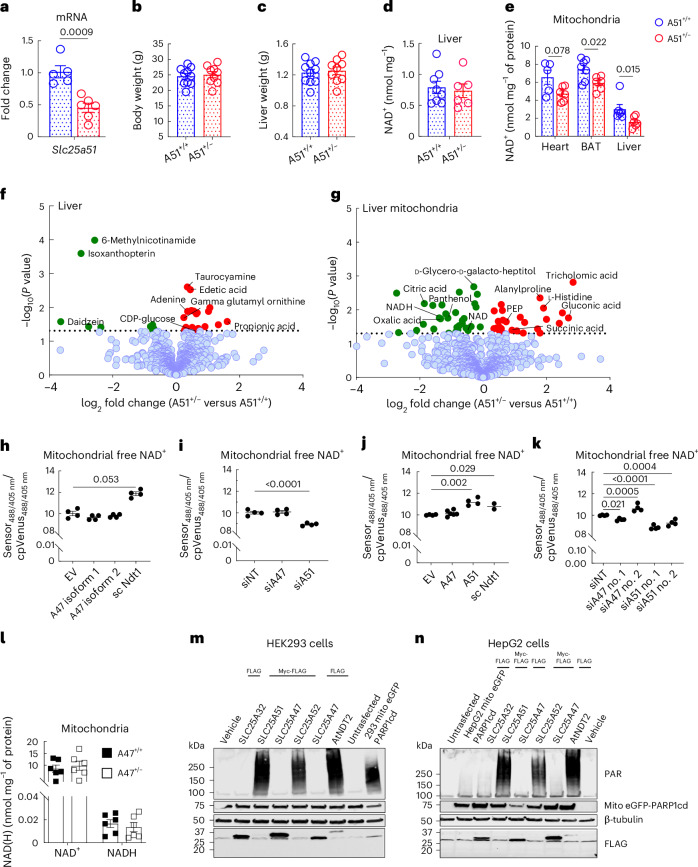
Slc25a51 haploinsufficiency alters mitochondrial NAD^+^ content in tissues including liver. *Slc25a51* heterozygous mice (A51^+/−^) are grossly normal. **a**, The *Slc25a51* mRNA expression in liver (*n* = 5 for A51^+/+^ and *n* = 6 for A51^+/−^). **b**,**c**, The body weights (**b**) and the liver weights (**c**), *n* = 10 for A51^+/+^ and *n* = 9 for A51^+/−^. **d**, The total hepatic NAD^+^ concentration in the liver (*n* = 8 for A51^+/+^ and *n* = 6 for A51^+/−^). **e**, The NAD^+^ content in the mitochondria isolated from the heart (*n* = 5 for A51^+/+^ and *n* = 6 for A51^+/−^), brown adipose tissue (BAT; *n* = 7 for A51^+/+^ and *n* = 6 for A51^+/−^) and livers (*n* **=** 6 for A51^+/+^ and *n* **=** 6 for A51^+/−^). **f**,**g**, The volcano plots showing changes in the metabolomic profiles of whole liver tissue (**f**) and liver mitochondria (**g**) in the A51^+/−^ mice (for LC–MS, *n* = 6–7 mice per group). The significantly changed metabolites were determined by *P* < 0.05 from an unpaired two-tailed Student’s *t*-test. **h**, The steady-state free mitochondrial NAD^+^ levels in HeLa cells overexpressing *SLC25A47* isoforms (isoform 1 and isoform 2) and *Saccharomyces cerevisiae* NDT1 (yeast mitochondrial NAD^+^ transporter), as measured using a mitochondrially targeted cpVenus NAD^+^ biosensor. **i**, The free mitochondrial NAD^+^ was measured in HeLa cells transfected with siRNA targeting human *SLC25A47*, *SLC25A51* or nontargeting scramble controls (*n* = 5 for all groups). **j**, The mitochondrial NAD^+^ in HepG2 cells overexpressing of *SLC25A47*, *SLC25A51* and *NDT1* (*n* = 4 for empty vector (EV); *n* = 6 for A47; *n* **=** 4 for A51 and *n* = 2 for sc Ndt1). **k**, The mitochondrial NAD^+^ in HepG2 cells transfected with siRNA targeting human isoforms of *SLC25A47*, *SLC25A51* or nontargeting scramble controls (*n* = 4 for all groups). **l**, The NAD^+^ and NADH content of mitochondria isolated from the livers of *Slc25a47*^*+/+*^ and *Slc25a47*^*−/−*^ mice (*n* = 6 for all groups). **m**,**n**, The immunoblots showing PAR-immunoreactive proteins as readouts for mitochondrial NAD^+^ availability in HEK293 (**m**) and HepG2 (**n**) cells expressing mitoPARP1cd and the indicated mitochondrial carrier proteins. For the knockdown and overexpression experiments in Hela and HepG2 cells, *n* = 4–6 biological replicates. The data are represented as mean, and the error bars represent s.e.m. The individual *P* values are shown where significant (*P* ≤ 0.05). The tests for normality and equality of variances, along with the resulting statistical test selected for each panel are provided in the statistical source data. Source data

**Extended Data Fig. 1:**
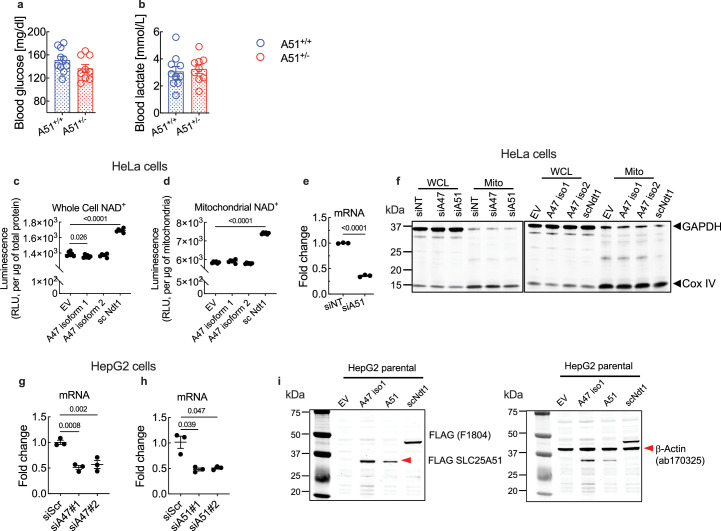
SLC25A47 knockdown or overexpression has no impact on mitochondrial NAD+ levels. **a**-**b**, Blood glucose (**a**) and lactate (**b**) measurements (n = 10 for A51^+/+^ and n = 9 for A51^+/-^). **c**-**d**, Whole cell (**c**) and mitochondrial (**d**) NAD^+^ measured in HeLa cells using a luminescence assay (n = 6 for all groups). **e**, mRNA validation of siRNA knockdown of *Slc25a51* (n = 3 for all groups). **f**, Western blot showing enrichment of Cox IV (mitochondrial) and depletion of GAPDH (cytosolic) in mitochondrial fractions isolated from HeLa cells. **g**-**h**, mRNA validation of siRNA knockdown of *Slc25a47* (**g**) and *Slc25a51* (**h**) in HepG2 cells (n = 3 for all groups). **i**, Western blot showing overexpression of SLC25A51 and SLC25A47 in HepG2 cells. Data is presented as the mean and error bars represent s.e.m. Individual *P* values are shown where significant (*P* ≤ 0.05). Tests for normality and equality of variances, along with the resulting statistical test selected for each panel are provided in statistical source data. Source data

To test whether SLC25A51 determines mitochondrial NAD^+^ content in vivo, as it does in cultured cells^11^, we initially took advantage of a null allele (created accidentally when the entire coding region was deleted during an attempt to modify the locus by CRISPR–Cas9 editing). Mice with two copies of this allele were never obtained from crosses of heterozygous parents (>200 pups, 25% expected), indicating that *Slc25a51* is an essential gene, with homozygous loss causing embryonic lethality. Despite a ~50% decrease in *Slc25a51* mRNA in liver (Fig. 1a), heterozygotes appeared unremarkable, with normal body weights, liver weights, blood glucose and blood lactate (Fig. 1b,c and Extended Data Fig. 1a,b). Although the total hepatic NAD^+^ content was unaltered, mitochondria isolated from several tissues of male mice, including the liver, had significantly reduced NAD^+^ concentrations (Fig. 1d,e), supporting the model that SLC25A51 is limiting for mitochondrial NAD^+^ uptake in vivo. Metabolomic analyses from whole liver and isolated mitochondria using liquid chromatography–mass spectrometry (LC–MS) confirmed that heterozygosity for *Slc25a51* was sufficient to lower the mitochondrial content of NAD^+^ and multiple related metabolites (Fig. 1f,g).

The lower mitochondrial NAD^+^ content in the livers of heterozygotes was especially interesting, given the recent suggestion that SLC25A47—and not SLC25A51—might be the major mitochondrial NAD^+^ transporter in hepatocytes^17^. We therefore performed a series of studies to test the potential NAD^+^ transport activity of SLC25A47. We were unable to detect an effect on mitochondrial NAD^+^ concentration using a fluorescent reporter when either of two isoforms of SLC25A47 was overexpressed in HeLa cells (Fig. 1h). Similarly, small interfering RNA (siRNA) against *SLC25A51*—but not *SLC25A47*—significantly lowered the mitochondrial NAD^+^ in HeLa cells (Fig. 1i), with the caveat that the expression of *SLC25A47* was below the limit of detection to begin with (Extended Data Fig. 1e). In HepG2 human hepatoma cells that have endogenous *SLC25A47*, the mitochondrial NAD^+^ content was similarly unaltered by SLC25A47 overexpression (Fig. 1j) and consistently decreased by the silencing of *SLC25A51* but not *SLC25A47* (Fig. 1k). Next, we isolated mitochondria from the livers of hepatocyte-specific *Slc25a47*-knockout mice^18^ and quantified NAD^+^ and NADH levels. Consistent with the results from cells, we did not detect any change (Fig. 1l). Finally, we assessed NAD^+^ transport based on poly-ADP-ribose (PAR) formation in cells expressing a mitochondrially targeted PARP1 catalytic domain (mitoPARP1cd; Fig. 1m,n). In a 293-derived cell line with a stable expression of mitoPARPcd1 and the endogenous *SLC25A51* knocked out, the overexpression of the mitochondrial NAD^+^ transporters SLC25A51, SLC25A52 or AtNDT2 resulted in strong PAR immunoreactivity, indicative of ongoing NAD^+^ import into mitochondria (Fig. 1m). Similarly, the cotransfection of these NAD^+^ transporters along with mitoPARP1cd in otherwise wild-type HepG2 cells gave a large increase in PAR signal (Fig. 1n). By contrast, the PAR signal in SLC25A47-expressing cells was unaffected and was comparable to that of cells expressing SLC25A32, which serves as a negative control. Our data are consistent with recent studies demonstrating lower mitochondrial NAD^+^ after the knockdown of SLC25A51^19^ but not SLC25A47^20^ in the liver. Therefore, SLC25A51—but not SLC25A47—is a major determinant of mitochondrial NAD^+^ content in hepatocytes, as in other cell types.

**Fig. 2:**
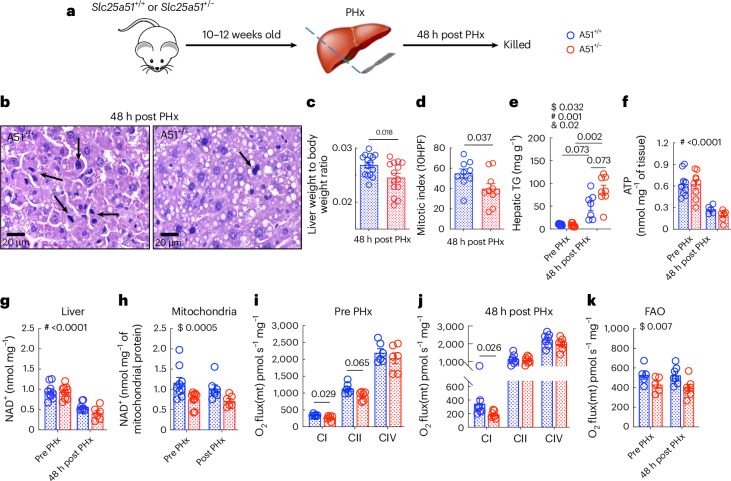
Partial loss of SLC25A51 impairs liver regeneration and mildly decreases mitochondrial respiratory capacity. Male *Slc25a51*^*+/−*^ mice and wild-type littermates were subjected to two thirds PHx and analysed 48 h later. **a**, A schematic of the experimental design. **b**, The representative sections of regenerating livers stained with H&E. The mitotic figures are marked by black arrows. **c**,**d**, The liver-to-body-weight ratios (*n* = 15 for A51^+/+^ and *n* = 14 for A51^+/−^) (**c**) and mitotic index (*n* = 9 for A51^+/+^ and *n* = 10 for A51^+/−^) (**d**) at 48 h post PHx. **e**,**f**, The hepatic TG (**e**) and ATP (**f**) content pre and 48 h post hepatectomy (*n* = 9 for pre in both genotypes; *n* = 7 for A51^+/+^ TG and *n* = 5 for A51^+/+^ ATP post PHx; *n* = 8 for A51^+/−^ TG and *n* = 8 for A51^+/−^ ATP post PHx). **g**,**h**, The hepatic NAD^+^ (**g**) and liver mitochondrial NAD^+^ (**h**) content pre and 48 h post injury (*n* = 9 for pre in both genotypes; *n* = 8 for A51^+/+^ liver and *n* = 7 for A51^+/+^ mito post PHx; *n* = 6 for A51^+/−^ for both liver and mito post PHx). **i**–**k**, The oxygen consumption flux in isolated mitochondria from resected (*n* = 6 for all groups) (**i**) and regenerated livers (*n* = 7 for all groups) (**j**) using complex I (CI, pyruvate), complex II (CII, succinate) and complex IV (CIV, TMPD-ASC)-dependent substrates or palmitoyl carnitine to measure fatty acid oxidation (FAO) (**k**) in A51 heterozygous mice and wild-type littermates (*n* = 5 for pre and *n* = 7 for post in both genotypes). The data are presented as the mean, and the error bars represent s.e.m. The ANOVA results are shown as main effects of genotype ($), time ($) and interaction (&). The individual *P* values are shown where significant (*P* ≤ 0.05). The tests for normality and equality of variances, along with the resulting statistical test selected for each panel, are provided in the statistical source data. 10HPF, ten high-power fields; O_2_ flux(mt), mitochondrial oxygen consumption flux. Source data

As the phenotype of *Slc25a51*^*+/−*^ mice was relatively mild, we next sought to challenge the mice with a metabolic stress. Liver regeneration is an energetically intensive process that has long been known to lower hepatic NAD^+^ levels^21–23^. We and others have shown that providing supplemental NAD^+^ precursors restores NAD^+^, improves bioenergetics and accelerates recovery after a PHx^14,15^. Conversely, blocking NAD^+^ synthesis in hepatocytes decreases regenerative capacity and causes energetic stress^14^. As *Slc25a51*^*+/−*^ mice have lower mitochondrial NAD^+^, but exhibit no change in total tissue NAD^+^ (Fig. 1d,e), we used this system to test whether it is the mitochondrial pool that is critical to support regeneration (Fig. 2a).

**Extended Data Fig. 2:**
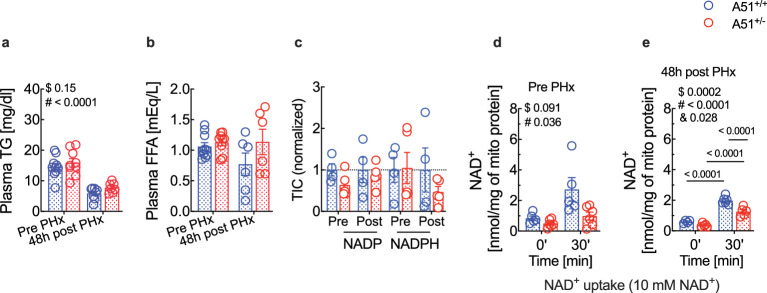
Partial loss of SLC25A51 in the liver influences lipid metabolism and decreases ex vivo mitochondrial NAD^+^ uptake. **a**-**b**, Circulating TG (**a**, n = 10 for A51^+/+^ pre and n = 8 post; n = 9 for A51^+/-^ pre and n = 7 for post) and FFA (**b**, n = 10 for A51^+/+^ pre and n = 6 post; n = 9 for A51^+/-^ pre and n = 6 for post) at pre and 48 h post PHx. **c**, NADP(H) analysed by LC-MS from whole liver tissue pre and 48 h post PHx (n = 4 for A51^+/+^; and n = 5 for A51^+/-^). **d**-**e**, NAD^+^ uptake assay from liver mitochondria isolated pre (**d**, n = 5 for A51^+/+^ and n = 6 for A51^+/-^) and 48 h post PHx (**e**, n = 4 for 0’ and n = 6 for 30’ in A51^+/+^; n = 5 for A51^+/-^ for both 0’ and 30’), based on NAD^+^ content before and after 30 minutes of incubation with 10 mM NAD^+^. Data is presented as the mean and error bars represent S.E.M. ANOVA results are shown as main effects of genotype ($), time ($), and interaction (&). Individual p-values are shown where significant (*P* ≤ 0.05). Tests for normality and equality of variances, along with the resulting statistical test selected for each panel are provided in statistical source data. Source data

Livers from *Slc25a51*^*+/−*^ mice regenerated more slowly than those of their wild-type littermates (Fig. 2b), displaying lower liver weight, less hepatocyte replication (Fig. 2c,d) and a trend toward more triglyceride (TG) accumulation (Fig. 2e). The hepatic ATP content was significantly reduced during regeneration irrespective of the genotype (Fig. 2f). The circulating TG were lower at 48 h of regeneration, but neither TG nor FFA were significantly affected by the *Slc25a51*^*+/−*^ genotype (Extended Data Fig. 2a,b). The total liver NAD^+^ concentration was unchanged by genotype but lower in both *Slc25a51*^*+/−*^ heterozygous mice and wild-type controls by 48 h post injury (Fig. 2g). The mitochondrial NAD^+^ levels were lower in the livers of *Slc25a51*^*+/−*^ mice and not significantly affected by regeneration (Fig. 2h). The NAD^+^ uptake was also significantly lower in mitochondria isolated from *Slc25a51*^*+/−*^ mice after PHx (Extended Data Fig. 2d,e). The mitochondrial respiration was slightly impaired in mitochondria isolated from livers of *Slc25a51*^*+/−*^ mice. This primarily affected complex I-dependent respiration and fatty acid oxidation (Fig. 2i-k), but we also detected a trend toward a reduction in complex II-dependent respiration in mitochondria isolated from *Slc25a51*^*+/−*^ livers before injury (Fig. 2i). This was unexpected as only complex I accepts electrons directly from NADH, and no effect on complex II was apparent post regeneration. Overall, these data are consistent with the model that hepatocyte mitochondrial NAD^+^ is limiting for liver regeneration but have constraints in that they were obtained in the context of a whole-body manipulation. Moreover, many interventions that compromise energetics could impair regeneration, but improving it provides more compelling evidence that a process is physiologically relevant.

**Fig. 3:**
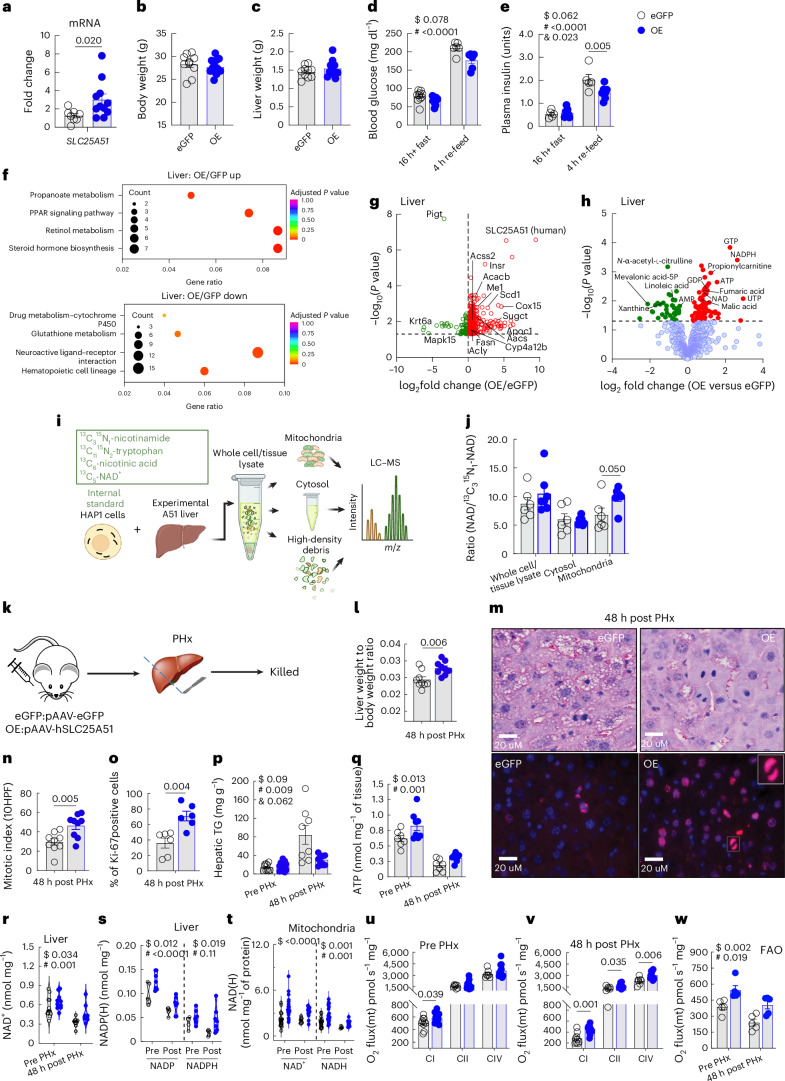
Overexpression of SLC25A51 in hepatocytes increases mitochondrial NAD^+^, promotes liver regeneration and maintains mitochondrial respiratory capacity post injury. Male mice were infected with AAV-expressing human SLC25A51 (OE) or eGFP under the control of the TBG promoter and studied 3–4 weeks post infection. **a**, The total *SLC25A51* transcript in liver (primers designed in a homologous region of human and mouse coding sequence (CDS)), (*n* = 8 for eGFP and *n* = 11 for A51 OE). **b**,**c**, The body weights (**b**, *n* = 10 for eGFP and *n* = 12 for A51 OE) and the liver weights (**c**, *n* = 10 for eGFP and *n* = 11 for A51 OE). **d**,**e**, The fasting and refed blood glucose (**d**, *n* = 10 for fasting GFP and *n* = 13 for fasting A51 OE; *n* = 6 for refed for both eGFP and A51 OE) and insulin (**e**, *n* = 5 for GFP and *n* = 7 for A51 OE) levels. **f**, The enrichment analysis of the liver transcripts. **g**,**h**, The untargeted proteomics (**g**) and metabolomics (**h**) profiling from liver tissue. The changes with a nominal *P* value less than 0.05 are coloured in red (upregulated) or in green (downregulated). For untargeted proteomics profiling, *n* = 3–6 per group, LC–MS experiments for metabolomics, *n* = 6–8 per group. **i**, The subcellular fractionation with stable isotope labelling by NAD-SILEC scheme in HAP1 cells **j**, The adjusted NAD^+^ concentrations in cellular compartments by NAD-SILEC (*n* = 6 for eGFP and *n* = 7 for A51 OE). The data for the mitochondrial fraction were analysed using a one-tailed *t*-test. Two-tailed *t*-tests were performed for the other subcellular fractions. **k**, The SLC25A51-OE and eGFP male mice were subjected to two thirds PHx and analysed 48 h later. **l**, The liver-to-body-weight ratio 48 h post PHx (*n* = 10 for eGFP and *n* = 11 for A51 OE). **m**, The representative regenerating liver sections stained in H&E and Ki-67 at 40× showing mitotic figures (in white arrows) and micro- and macrovesicular fatty changes. **n**, The mitosis as determined by counting mitotic figures in hepatocytes under high power in H&E sections (*n* = 9 for both eGFP and A51 OE). **o**, The percent of Ki-67 positively stained cells (*n* = 6 for both eGFP and A51 OE). **p**, The hepatic TG content (*n* = 10 for eGFP pre and *n* = 8 for post PHx; *n* = 13 for A51 OE pre and *n* = 9 for post PHx). **q**, The hepatic ATP content (*n* = 7 for eGFP, *n* = 8 for A51 OE pre and *n* = 7 for post PHx). **r**,**s**, The total liver NAD^+^ (*n* = 10 for eGFP pre and *n* = 9 for post PHx; *n* = 13 for A51 OE pre and *n* = 8 for post PHx) (**r**) and NADP(H) (*n* = 4–5 for eGFP and *n* = 6–7 for A51 OE) (**s**) content. **t**, The mitochondrial NAD^+^ and NADH content (*n* = 21 for eGFP pre NAD(H); *n* = 22 and 21 for A51 OE NAD(H), respectively; *n* = 8 and 4 for eGFP NAD(H) post, respectively; *n* = 11 and 7 for A51 OE NAD(H) post, respectively). The violin plots show individual data points and the median. **u**–**w**, The state three-coupled respiration in mitochondria from resected (**u**) and regenerated (**v**) livers, using complex I (CI, pyruvate), complex II (CII, succinate) and complex IV (CIV, TMPD-ASC)-dependent substrates (*n* = 9–10 for CI, *n* = 7 for CII and CIV in eGFP; *n* = 11 for A51 OE). **w**, The mitochondrial oxygen consumption using fatty acid oxidation substrates (*n* = 5 for both genotypes). The data are presented as the mean, and the error bars represent s.e.m. The ANOVA results are shown as the main effects of genotype ($), time ($) and interaction (&). The individual *P* values are shown where significant (*P* ≤ 0.05). The tests for normality and equality of variances, along with the resulting statistical test selected for each panel, are provided in the statistical source data. 10HPF, ten high-power fields; O_2_ flux(mt), mitochondrial oxygen consumption flux. Panel **i** created with BioRender.com. Source data

**Extended Data Fig. 3:**
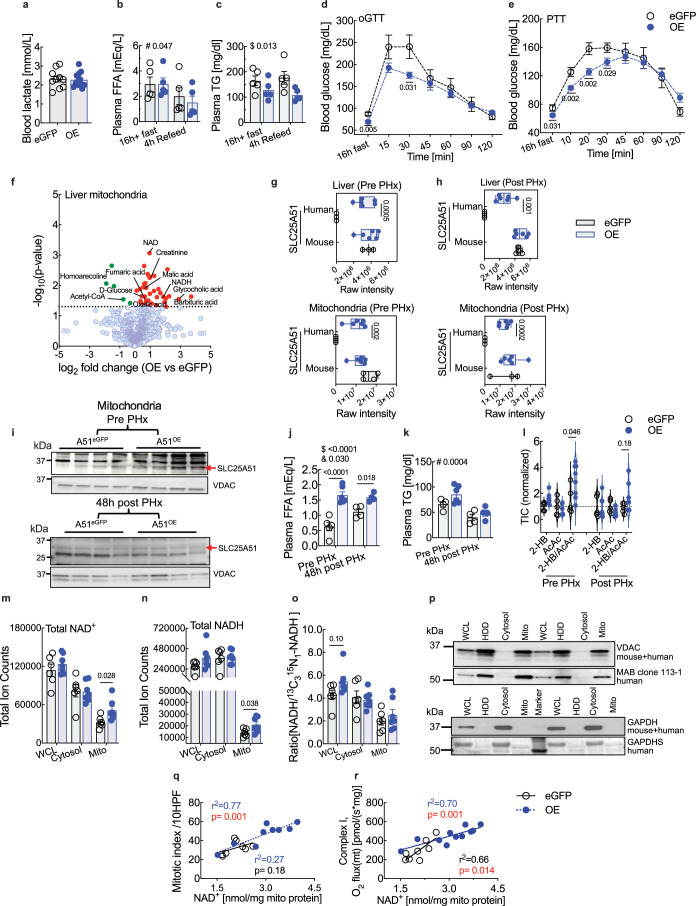
SLC25A51 overexpression drives changes in metabolism and increases in mitochondrial NAD^+^ that correlate with Complex I-dependent respiration. **a**, Blood lactate levels (n = 10 for eGFP and n = 12 for A51 OE). **b**-**c**, Plasma FFA (**b**) and TG (**c**) at fasting or after refeeding (n = 5 for both eGFP and A51 OE). **d**-**e**, Oral glucose tolerance test [oGTT] (**d**, n = 5 for eGFP and n = 6 for A51 OE) and pyruvate tolerance test [PTT] (**e**, n = 9 for eGFP and n = 12 for A51 OE). **f**, Metabolomic profiling of liver mitochondria (n = 5-8 per group). **g**-**h**, Liver and mitochondrial SLC25A51 protein expression by proteomics analyses at pre (**g**) and 48 h post regeneration (**h**). Whisker plots show the interquartile range and whiskers represent minimum to maximum values (n = 3-4 for eGFP and n = 6 for A51 OE). **i**, Immunoblots from isolated liver mitochondria from pre and 48 h post PHx probed for SLC25A51 protein. **j**-**l**, Circulating FFA (**j**, n = 5 for eGFP pre and n = 4 for post; n = 5 for A51 OE pre and n = 4 for post), TG (**k**, n = 4 for eGFP; n = 6 for A51 OE pre and n = 4 for post), and ketones (**l**, n = 5 for eGFP and n = 8 for A51 OE) at pre and 48 h post PHx. Violin plot shows individual data points and the median. **m**-**o**, Total NAD^+^ (**m)** and NADH (**n**) levels as well as adjusted NADH from NAD-SILEC (**o**), (n = 6 for eGFP and n = 7 for A51 OE). **p**, Validation of subcellular fractionation by immunoblots (n = 3 per group). **q**-**r**, Positive correlations between mitotic indices (**q**) or complex-I dependent respiration (**r**) and mitochondrial NAD^+^ concentration in regenerating livers from SLC25A51-overexpressing or control mice (n = 8 for eGFP and n = 11 for A51 OE). Data was adjusted to a linear fit and the Pearson’s correlation coefficient is presented along with the p-value for the slope. Data are presented as the mean and error bars represent S.E.M. ANOVA results are shown as main effects of genotype ($), time ($), and interaction (&). Individual p-values are shown where significant (p ≤ 0.05). Tests for normality and equality of variances, along with the resulting statistical test selected for each panel are provided in statistical source data. Source data

**Extended Data Fig. 4:**
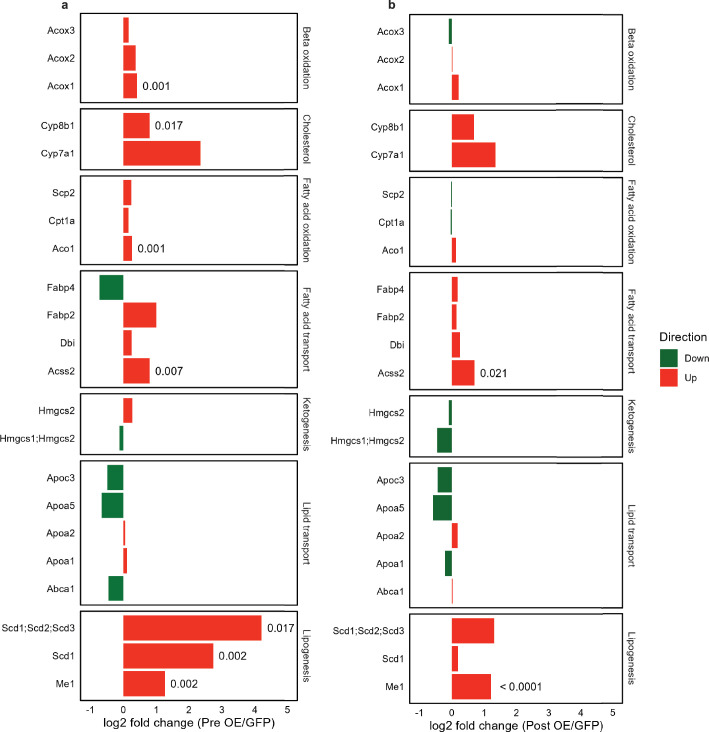
SLC25A51 overexpression affects expression of PPARα targets involved in lipid metabolism. Proteomic profiling of liver tissue from SLC25A51-overexpressing and eGFP control mice showing protein expression of PPARα targets. **a**-**b**, Effects of SLC25A51 overexpression pre (**a**) and 48 h post (**b**) partial hepatectomy. Statistical analysis was performed by an unpaired two-tailed Student’s *t*-test (n = 3 mice for eGFP control and n = 6 for A51 OE mice). Individual *P* values are shown where significant (*P* < 0.05). Source data

**Extended Data Fig. 5:**
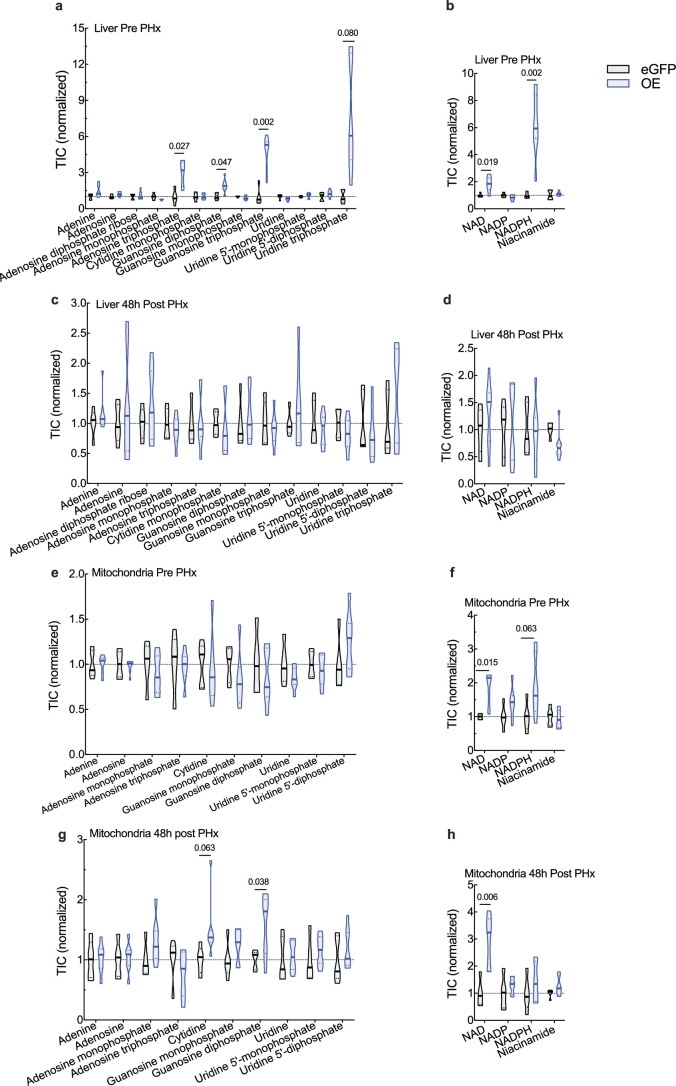
SLC25A51 overexpression increases nucleotide triphosphates and NAD^+^-related metabolites. **a**-**d**, Nucleotides and NAD^+^ metabolites from livers pre (**a** and **b**) and 48 h post (**c** and **d**) partial hepatectomy detected by LC-MS. **e**-**h**, Nucleotides and NAD^+^ metabolites from isolated liver mitochondria pre (**e** and **f**) and 48 h post (**g** and **h**) partial hepatectomy. Violin plots show the distribution of the data points and the median. Statistical analyses were performed by a two-tailed Student’s *t*-test (n = 5 mice for eGFP control and n = 6-8 for A51 OE). Individual *p*-values are shown where significant (*P* < 0.05). Source data

To test the hypothesis that increasing mitochondrial NAD^+^ in a cell-type specific manner could improve regeneration, we used adeno-associated virus (AAV) expressing human SLC25A51 under the control of the hepatocyte-specific *Serpina7* promoter. The total *SLC25A51* transcripts (Fig. 3a) and protein (Extended Data Fig. 3g,i) in liver were increased approximately two- to three-fold. This intervention did not cause obvious behavioural changes or affect body or liver weight (Fig. 3b,c), although it consistently caused a modest decrease in blood glucose (Fig. 3d) but not lactate (Extended Data Fig. 3a) levels. The plasma TG levels were lower in the SLC25A51-overexpressing mice, and insulin was significantly lower in the refed state (Fig. 3e and Extended Data Fig. 3c). The lower glucose levels were maintained during glucose and pyruvate tolerance tests (Extended Data Fig. 3d,e). The transcript and protein profiling revealed an upregulation in PPAR signalling and factors related to fatty acid biosynthesis and metabolism (Fig. 3f,g). This included a striking upregulation of Cyp4a12b and short-chain dehydrogenases involved in retinol metabolism and a general increase in PPARα targets involved in lipid metabolism and fatty acid biosynthesis (Extended Data Fig. 4a,b). The metabolomic profiling of mitochondria confirmed the expected increase in mitochondrial NAD^+^ (Extended Data Fig. 3f). The profiling of the whole tissue revealed a significant increase in ATP and in other nucleotide triphosphates in the liver, suggesting a higher energy charge when mitochondrial NAD^+^ content is increased (Extended Data Fig. 5a,b,f). As there was a small increase in total liver tissue NAD^+^ (Fig. 3h), it was unclear whether cytosolic NAD^+^ was decreased as a result of mitochondrial uptake or whether cytosolic NAD^+^ was maintained. To directly test this, we performed a fractionation experiment with stable isotope labelling by essential nutrients in cell culture (SILEC)^24^ (Fig. 3i). In this technique, samples are spiked with a constant number of cells in which the compounds of interested are fully labelled with heavy isotopes. The ratio of unlabelled to labelled compounds accounts for variations in handling and fractionation. Using this technique, we confirmed the increase in mitochondrial NAD^+^ in SLC25A51-overexpressing livers and were unable to detect any change in cytosolic NAD^+^ (Fig. 3j and Extended Data Fig. 3m–o).

Next, we subjected the SLC25A51-overexpressing mice to the two thirds PHx protocol to study its influence on liver regeneration (Fig. 3k). In contrast to the livers with low mitochondrial NAD^+^, the livers from the mice overexpressing SLC25A51 regenerated significantly better than those of the enhanced green fluorescent protein (eGFP)-expressing controls (Fig. 3m). The liver weights and indices of hepatocyte proliferation including mitotic cells and Ki-67-positive cells were significantly increased by 48 h post injury (Fig. 3l–p). The decreases in lipid storage were readily apparent histologically (Fig. 3m), and the ATP levels declined during regeneration and were improved in SLC25A51-overexpressing livers (Fig. 3q). The liver tissue NAD^+^ and NADP^+^ levels declined during regeneration and were slightly increased by SLC25A51 overexpression (Fig. 3r,s). The mitochondrial NAD^+^ and NADH levels were elevated by SLC25A51 overexpression, as expected (Fig. 3t). In isolated mitochondria, small improvements in complex I-dependent respiration and fatty acid oxidation were detected, and these became clearer and extended to complex I-, II- and IV-dependent respiration in the regenerating tissue (Fig. 3u–w). Both hepatocyte replication rate (mitotic index) and complex I-dependent respiration were highly correlated with mitochondrial NAD^+^ content (Extended Data Fig. 3m,p).

**Fig. 4:**
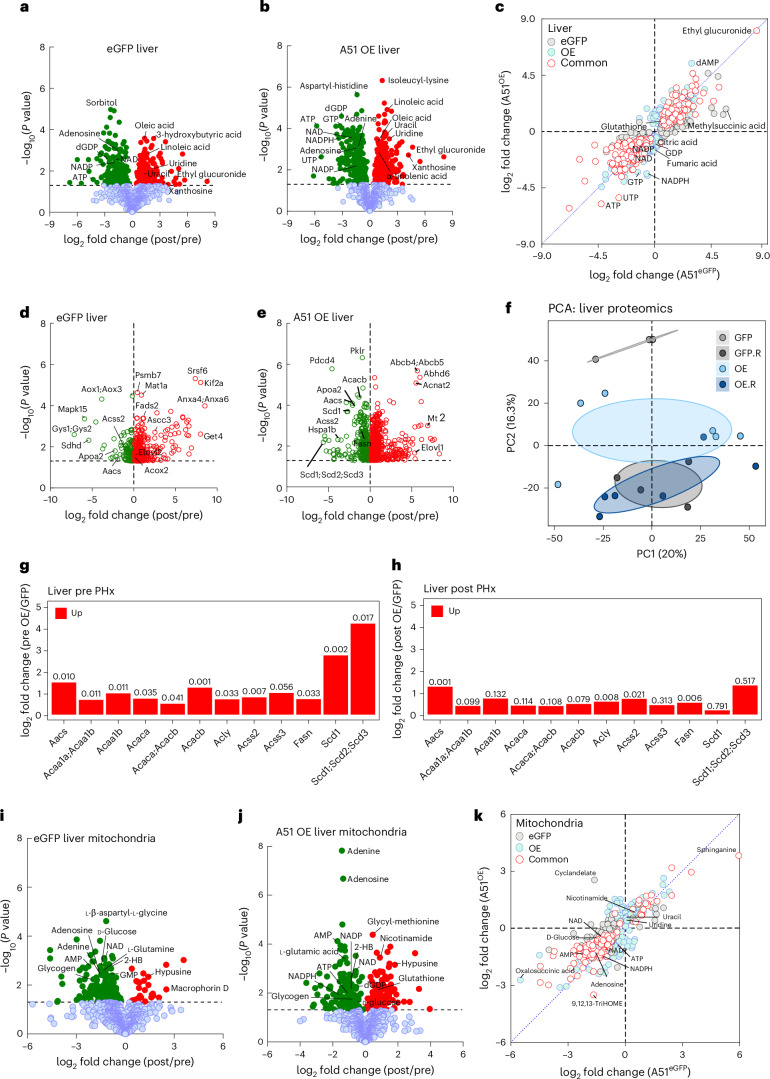
SLC25A51 overexpression promotes fatty acid metabolism and attenuates the depletion of mitochondrial metabolites during liver regeneration. The changes in metabolites and protein expression in livers and isolated hepatic mitochondria at 48 h post PHx in mice infected with control AAV (eGFP) or overexpressing SLC25A51 in hepatocytes (OE) are shown. **a**,**b**, The metabolite changes during regeneration in control (**a**) and SLC25A51-overexpressing (**b**) liver tissue. **c**, The correlation plots of log_2_ fold changes in liver. **d**,**e**, The protein changes during regeneration in control (**d**) and SLC25A51-overexpressing (**e**) liver tissue. **f**, The principal component analysis (PCA) of liver proteomes showing principal components 1 and 2 (PC1, PC2). GFP.R and OE.R indicate the regenerated livers. **g**,**h**, The expression of proteins involved in fatty acid biosynthesis in SLC25A51-overexpressing resected (**g**) and regenerated (**h**) livers. **i**,**j**, The metabolite changes in mitochondria during regeneration of control (**i**) and SLC25A51-overexpressing (**j**) livers. **k**, The correlation plots of log_2_ fold changes in the metabolites of isolated liver mitochondria. The volcano plots show metabolites (in closed circles) and proteins (in open circles) that were significantly changed with a *P* value less than 0.05 coloured in red (upregulated) or in green (downregulated). The correlation plots of log_2_ fold change in OE versus eGFP were made by selecting all significant metabolites within the eGFP comparison alone, OE comparison alone or shared (common) for both liver tissue and isolated mitochondria. The metabolites and proteins that were significantly changed with a *P* value less than 0.05 were determined by two-tailed Student *t*-tests. The individual *P* values are shown where significant (*P* < 0.05) (*n* = 3–5 mice per group for eGFP control and A51 OE, *n* = 6–8 mice per group). Source data

**Extended Data Fig. 6:**
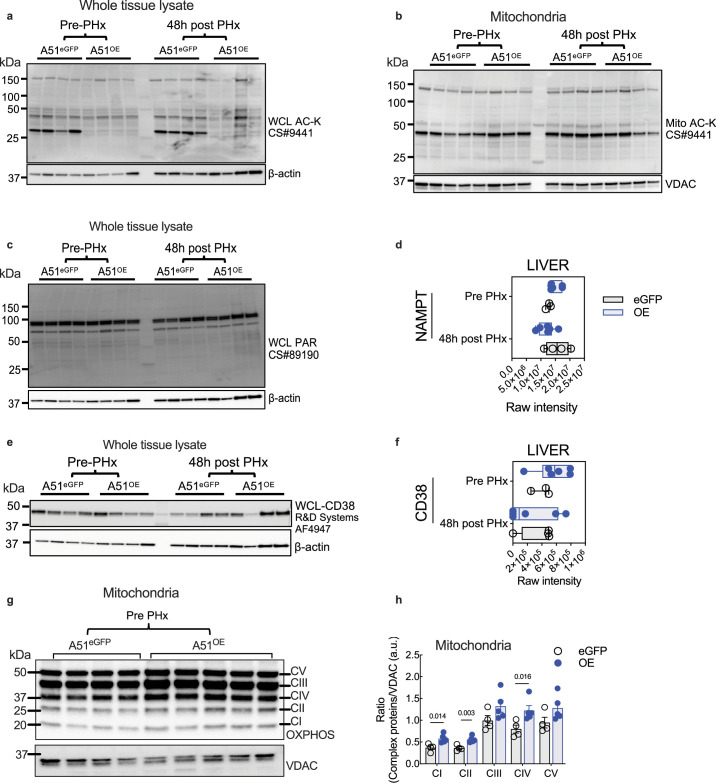
Effects of SLC25A51 overexpression on acetylation, PARylation, and expression of oxidative phosphorylation complexes in liver. **a-b**, Immunoblots from whole liver tissue lysates (**a**) and isolated mitochondria (**b**) probed for acetylated lysine (Ac-K). **c**, Total PARylation blot from whole tissue lysates. **d**, Liver NAMPT protein expression by proteomics analyses pre and 48 h post regeneration. **e-f**, Immunoblot from liver tissue lysates probed for CD38 (**e**) proteomics analyses of CD 38 from liver tissue (**f**). **g**-**h**, Immunoblot (**f**) and quantification (**g**) of protein complexes in the electron transport chain from isolated mitochondria. (n = 4 mice for eGFP control and n = 5 for A51 OE mice). Proteomics whisker plots show the interquartile range and whiskers represent minimum to maximum values (n = 3-4 for eGFP and n = 6 for A51 OE). Error bars in bar plots represent s.e.m. Individual *P* values are shown where significant (*P* ≤ 0.05). Source data

**Extended Data Fig. 7:**
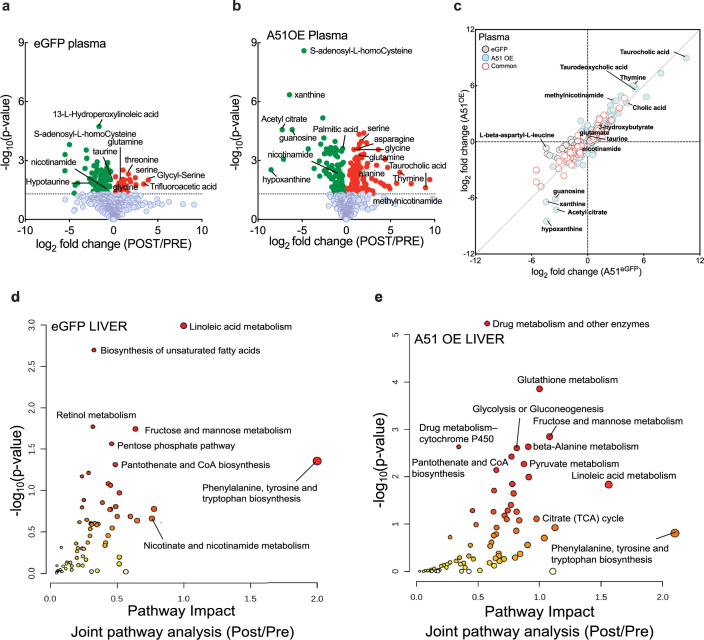
SLC25A51 overexpression influences the plasma metabolome during liver regeneration. Metabolomic profile of plasma acquired from SLC25A51 overexpressing (OE) and eGFP control mice pre and 48 h post hepatectomy. **a**-**b**, Depleted and enriched metabolites during regeneration in eGFP (**a**) and A51 overexpressing (**b**) mice. Significant metabolites were determined by p < 0.05 from an unpaired two-tailed Student’s t-test. **c**, Correlation plots of log2 fold change were plotted from plasma by selecting all significant metabolites within the eGFP comparison alone, OE comparison alone or a shared (common) subset. **d**-**e**, Joint pathway analysis of significant metabolites and proteins (p < 0.05 from Student’s *t*-test) from liver tissue of eGFP controls (**d**) and SLC25A51 overexpressing (**e**) livers using MetaboAnalyst 6.0^45^, (n = 5 mice for eGFP control and n = 6-8 for A51 OE). Source data

**Extended Data Fig. 8:**
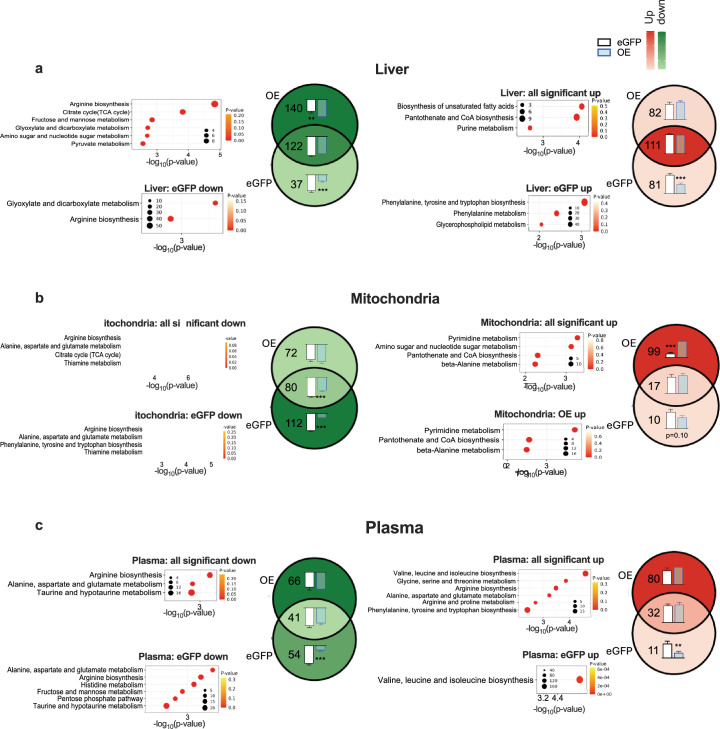
Enrichment analysis of metabolite changes during liver regeneration with or without overexpression of SLC25A51. Untargeted metabolomics datasets were derived from liver tissue (**a**), isolated hepatic mitochondria (**b**), and plasma (**c**) pre and 48 h post hepatectomy from SLC25A51 overexpressing and eGFP control mice. Statistical comparisons to obtain significantly regulated metabolites were made between regenerated versus resected states for OE and eGFP independently. Venn diagrams show significantly downregulated (green) or upregulated (red) metabolites for each group. Bar plots in Venn diagrams show the mean log2 fold change of the regenerated versus resected normalized counts for all metabolites in the intersect or individual subsets [either OE or GFP], error bars represent S.E.M. The number of metabolites in each subset is denoted in the figure. Statistical analysis for the bar plots was performed using an unpaired Student’s *t*-test with *P* values represented as *P* ≤ 0.05 (*), *P* ≤ 0.01 (**), and *P* ≤ 0.0001 (***), (n = 5 for eGFP and n = 8 for A51 OE). Enrichment pathway scatter plots were made by selecting the metabolites within each subset, eGFP alone, OE alone, or all significant up- or downregulated metabolites as indicated. Source data

**Extended Data Fig. 9:**
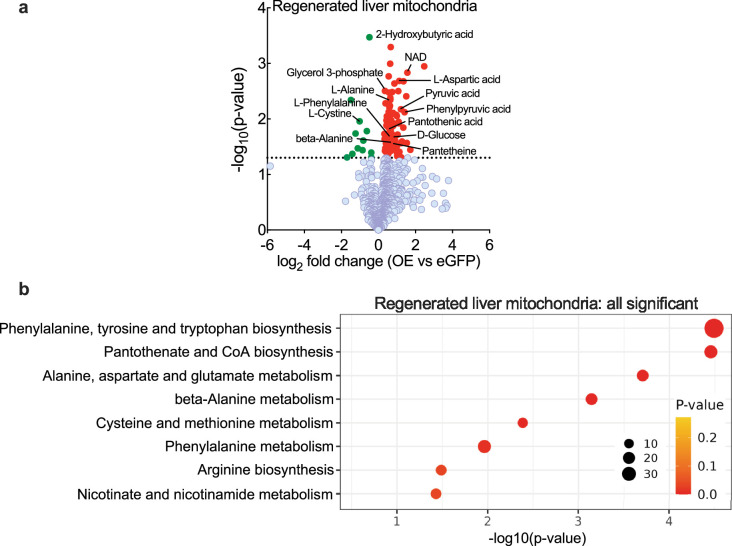
SLC25A51 overexpression is associated with changes in amino acid metabolism and pantothenate and CoA biosynthesis in mitochondria from regenerating livers. **a**, Altered metabolites in isolated mitochondria from SLC25A51 overexpressing vs. control livers during regeneration. Significant metabolites were determined by *p* < 0.05 from an unpaired two-tailed Student’s t-test. Metabolites with a *P* value less than 0.05 are colored in red (upregulated) or in green (downregulated). **b**, Pathway enrichment based on significantly altered metabolites (*P*  < 0.05 from Student’s *t*-test) in isolated mitochondria from regenerating SLC25A51 overexpressing livers vs. eGFP controls. Source data

To gain further insight into how mitochondrial NAD^+^ influenced the regenerative process, we performed metabolomic and proteomic profiling (Fig. 4). At the level of the whole liver, changes in metabolites induced by regeneration (regenerating livers compared with the corresponding resected livers) were quite similar between SLC25A51-overexpressing livers and eGFP controls (Fig. 4a–c). In fact, in several cases where the response to regeneration appeared altered, this was driven more by initial differences in the resected livers than by changes in the concentration in regenerating livers. For example, UTP and ATP decrease more in SLC25A51-overexpressing livers during regeneration but actually begin higher before injury (8-fold and nearly 3-fold, respectively) (Extended Data Fig. 5a) and remain higher or unchanged in the SLC25A51-overexpressing livers during regeneration (1.4-fold and 1.0-fold, respectively). Similarly, the proteomic analyses reveal a convergence during regeneration, with SLC25A51-overexpressing livers separating from those of eGFP controls along principal component 2 at resection but almost completely overlapping in the regenerated state (Fig. 4d–f). The SLC25A51-overexpressing livers did not display consistent changes in protein acetylation, PARylation or CD38 expression but had an increased expression of several electron transport chain components (Extended Data Fig. 6). The proteins involved in fatty acid biosynthesis were upregulated in the SLC25A51-overexpressing livers compared with eGFP controls at resection and remain higher 48 h post regeneration (Fig. 4g,h). The enrichment analysis (Metaboanalyst) of the decreased metabolites from both sets of regenerating livers implicated arginine biosynthesis and the tricarboxylic acid cycle, whereas increased metabolites in regenerating livers were enriched for the biosynthesis of unsaturated fatty acids and pantothenate and CoA biosynthesis (Extended Data Figs. 7 and 8a). The joint pathway analysis from proteomics and metabolomics confirmed an increase in retinol and fatty acid biosynthesis pathways (Extended Data Fig. 7d,e). In mitochondria isolated from control (eGFP) livers, regeneration was associated with loss of many metabolites (Fig. 4i,k). These effects were attenuated in mitochondria from SLC25A51-overexpressing livers (Fig. 4j and Extended Data Fig. 9), which further exhibited an increase in metabolites that had been unaffected in control livers (Fig. 4a). Thus, the lower mitochondrial NAD^+^ levels experienced during regeneration may not be sufficient to maintain fluxes through multiple metabolic pathways in mitochondria, which could contribute to energetic stress and limit the rate at which biomass can be created.

In plasma, taurine and hypotaurine metabolism were represented among the decreased metabolites during regeneration, and branched chain amino acid biosynthesis was highly represented among the increased metabolites (Extended Data Fig. 8c). As in mitochondria, more plasma metabolites were decreased than increased during regeneration, and a subset of these were maintained better in plasma from SLC25A51-overexpressing mice (Extended Data Fig. 7a–c). Interestingly, hypoxanthine, xanthine and guanosine, all substrates for purine salvage, were highly increased in the plasma of mice expressing liver-specific SLC25A51 at baseline (67-, 15- and 32-fold, respectively). All were decreased during regeneration, resulting in a larger fold-decrease for the SLC25A51-overexpressing mice (Extended Data Fig. 7a–c), despite still having modestly higher levels overall during regeneration (four-, four- and fivefold, respectively). These metabolites were not elevated in liver tissue, suggesting that it is not the source of the higher plasma levels. Overall, our results suggest that hepatocyte mitochondrial NAD^+^ levels have a strong influence on liver regeneration through mechanisms that include maintaining energy charge and mitochondrial metabolite levels while promoting lipid metabolism.

The finding that changing SLC25A51 expression is sufficient to cause corresponding changes in the hepatocyte mitochondrial NAD^+^ pool size as well as the regenerative capacity of the liver has several important implications. First, our work indicates that SLC25A51 is the major determinant of mitochondrial NAD^+^ in vivo for several tissues, including the liver, as was recently shown for aortic smooth muscle cells^25^ and as we and others have shown it to be for many cell types in culture^11–13^. This is important because it is the first demonstration that the loss of the transporter is not effectively compensated by other mechanisms in the liver, and because an alternative candidate, SLC25A47, has been proposed to transport NAD^+^ in hepatocytes^17^. Our data support SLC25A51 as the major mitochondrial NAD^+^ carrier in the liver (as also recently shown for smooth muscle cells in vivo^25^) and suggest that any effects of SLC25A47 on mitochondrial NAD^+^ pools may occur via indirect mechanisms^20^.

Second, our data implicate the hepatocyte mitochondrial NAD^+^ pool as a limiting factor for liver regeneration. Regeneration is known to cause a fall in tissue NAD^+^ levels, and we and others have previously shown that systemic supplementation with NAD^+^ precursors can improve liver regeneration^14–16^. The present results allow us to narrow down the likely mechanisms of action to a single subcellular pool in a single cell type. Although the present results involved only male mice, both sexes respond similarly to supplementation, and we hypothesize that SLC25A51 plays the same role in females. Importantly, we cannot completely rule out an NAD^+^-independent effect of SLC25A51. For example, this class of transporter frequently functions as an exchanger^26^, and a potential counter substrate for NAD^+^ might independently influence liver regeneration. However, we view the direct import of mitochondrial NAD^+^ as the most likely mechanism, given the evidence that increasing NAD^+^ through other mechanisms is similarly beneficial for liver regeneration^27^.

We found that modest changes in hepatocyte mitochondrial NAD^+^ have only minor phenotypic consequences under standard living conditions but can more dramatically affect the response to a stress such as liver regeneration. Consistent with the view that mitochondrial NAD^+^ becomes more important under stress, we have previously observed that mitochondria sequester NAD^+^ even as total tissue levels fall in the liver and kidneys during haemorrhagic shock^28^, and we show here that loss of NAD^+^ from mitochondria is less than that from bulk tissue during liver regeneration. In contrast to skeletal muscle, where mitochondria NAD^+^ appears to be in excess^29,30^, maximal respiratory capacity is improved by additional NAD^+^ in hepatocyte mitochondria. This increased capacity for mitochondrial fluxes and ATP generation may become crucial during intense metabolic stresses such as liver regeneration. In fact, it has recently been suggested that hepatocytes become metabolically inflexible during liver regeneration to force dependence on mitochondrial fatty acid oxidation as a selection for metabolically healthy cells^31^. That study further suggests that the activity of mitochondrial complex I is dispensable for hepatocyte replication. Although complex I-dependent respiration is clearly responsive to mitochondrial NAD^+^ levels in our data, it may be the roles of NAD^+^ in multiple anaplerotic processes and especially fatty acid oxidation that ultimately limit regeneration when mitochondrial NAD^+^ falls and that are improved by SLC25A51. Consistently, both mRNA- and protein-based analyses suggested increased lipid metabolism in the SLC25A51-overexpressing livers. Thus, higher mitochondrial NAD^+^ levels may promote increased lipid metabolism and thereby prime livers to better respond to the metabolic demands of regeneration.

Notably, there is evidence for at least the transient protection of the mitochondrial NAD^+^ pool from degradation by nuclear or cytosolic enzymes, which may be another benefit of importing NAD^+^ into the organelles under harsh conditions^32,33^. Mitochondrial NAD^+^ has further been implicated as a pool that can be released to support nuclear signalling in response to DNA damage; therefore, enhancing mitochondrial NAD^+^ content might prime cells to respond more robustly to damage^34^. In contrast to this prediction, the same group recently showed that the loss of SLC25A51 enhances nuclear DNA repair, ostensibly because NAD^+^ synthesis is maintained, resulting in higher nuclear and cytosolic concentrations when mitochondrial uptake is blocked^35^. The effect of SLC25A51 expression on nuclear NAD^+^ concentration could be relevant to the present results, given that PPARα targets are affected, and PPARα can be activated by the NAD^+^-dependent enzyme SIRT1^36^. Our data are most consistent with a compensatory increase in NAD^+^ synthesis to maintain cytosolic and nuclear levels in hepatocytes overexpressing SLC25A51. However, the ultimate effects on nuclear DNA repair and NAD^+^-dependent enzymes in that compartment are yet to be determined.

The finding that mitochondrial NAD^+^ can be the pool that is relevant to biological outcomes has important implications for studying NAD^+^ metabolism. As mitochondrial NAD^+^ can be changed drastically without a major influence on total cellular NAD^+^ content^11,12^, standard measures of NAD^+^ in tissues may be insufficient to detect mitochondrial NAD^+^ deficiency. Likewise, supplementation may increase total tissue NAD^+^ levels with marginal or no effects on mitochondrial levels. Moreover, other discrete pools of NAD^+^ exist, at least in peroxisomes, endoplasmic reticulum and the Golgi, and each may be important in a distinct set of conditions. These observations highlight the need to improve our understanding of the specific NAD^+^-dependent processes that are sensitive to physiologically relevant changes in its concentration, including the relevant cell types and subcellular compartments, to support translational efforts around NAD^+^ metabolism.

Altogether, these studies establish that SLC25A51 is the major determinant of the mitochondrial NAD^+^ pool in hepatocytes in vivo and implicate changes in this pool as the mechanism by which NAD^+^ precursors can support liver regeneration. Whether the mitochondrial pool is the relevant therapeutic target in other models where systemic NAD^+^ supplementation is therapeutic remains to be determined.

## Methods

### Mouse model generation and animal housing

All animal work was performed in accordance with the guidelines and approval of the University of Pennsylvania Institutional Animal Care and Use Committee (IACUC). The animals were housed in groups of four to five mice per cage in a pathogen-free barrier facility kept at ambient temperature in a 12-h light–dark cycle with free access to food and water.

### Generation of *Slc25a51*^*+/−*^ mice

A null allele for *Slc25a51*, lacking the entire coding region, was generated accidentally during an attempt to insert loxP sites at the A51 locus using CRISPR–Cas9. A failed repair template targeting the 5′ side of the translated region resulted in the deletion of exon 3, which contains the entire coding region, on one allele. This animal was crossed to the C57BL/6NJ background, and the heterozygous animals were then crossed.

### SLC25A51 overexpression

The 10–12-week-old male mice were injected retro-orbitally with 1 × 10^12^ genome copies (GC) of either custom-made AAV-expressing human SLC25A51 (*pAAV[Exp]TBG* > *hSLC25A51[*NM_033412.4*]/FLAG:IRES:EGFP:WPRE*) from Vector Builder (no. *AAV8LP[VB200511-1377vwe]-K2*) or ultrapurified eGFP (eGFP control, AAV8 virus) under the control of the *Serpina7* which encodes thyroxine-binding globulin (TBG) promoter. The mice were studied beginning at 3–4 weeks post infection.

### PHx

The male mice (12–16 weeks old) underwent PHx according to the protocol of Mitchell and Willenbring^37,38^ between 08:00 and 13:00. In brief, after isoflurane anaesthesia, a ventral midline incision was made. The median and left lateral lobes comprising 70% of the liver were surgically resected by pedicle ligation. The animals were killed at 48 h post PHx. The resected and regenerating livers were either freeze-clamped in liquid nitrogen for metabolite analysis, fixed in 4% paraformaldehyde or taken for mitochondrial isolation.

### Histological analysis

Hepatic steatosis or fatty changes were visualized, and mitotic indices were counted in haematoxylin and eosin (H&E)-stained paraffin-embedded liver sections (5-µm thickness). The mitotically active areas were first previewed under lower magnification and then counted in ten high-power fields (40× objective, 400× magnification) for the quantification of total mitotic figures. The representative photomicrographs were taken at 400× magnification using a light microscope (Olympus DP72) coupled with a digital image acquisition system.

For the immunofluorescent detection of Ki-67 expression in paraffin sections, the antigens were retrieved in Citrate buffer, followed by quenching and blocking in 3% hydrogen peroxide for 15 min. The slides were then incubated overnight at 4 °C in a humidified chamber with the Ki-67 (Abcam, ab16667) antibody, followed by ImmPRESS (Peroxidase) anti-rabbit secondary (Vector MP-740) for 60 min at room temperature. The slides were incubated in TSA tetramethylrhodamine reagent (Akoya Biosciences SAT702001EA) and counterstained in 4′,6-diamidino-2-phenylindole. The images were captured at 400× magnification using a Nikon Eclipse E600 fluorescence microscope equipped with Qimaging, Q click digital camera. QuPath software^39^ was used to quantify the Ki-67-positive cells. Approximately 300 cells were counted from each slide at 20×, and the per cent of Ki-67-positive cells were quantitated and normalized to the total cell number (*n* = 6 for both eGFP and OE).

### Glucose and insulin measurements

Oral glucose tolerance tests were performed on overnight-fasted mice, and blood glucose measurements were recorded using a glucometer (ARKRAY Glucocard Vital). The dextrose solution (20% w/v) in saline was gavaged at a dose of 2 g kg^−1^ body weight, and blood glucose was measured from tail blood after 0, 15, 30, 45, 60, 90 and 120 min. For fasting and refeeding experiments, blood glucose measurements were taken by a glucometer, and an additional 40 µl of tail blood was collected in a lithium heparin-coated microvette tube from a non-restrained mouse for insulin and lipid measurements. The blood was collected after an overnight fast and again after 4 h of refeeding. The blood samples were centrifuged at 8,000*g* for 10 min to separate plasma. The plasma insulin was measured in duplicate by double-antibody radio-immunoassays (Millipore). Where indicated, blood lactate measurements were also recorded from tail vein blood using the Nova Biomedical Lactate PLUS^M^ meter. Pyruvate tolerance testing was similarly performed on overnight-fasted mice at a dose of 1 g kg^−1^ (intraperitoneally), body weight, with blood glucose and lactate monitored from tail blood at 0, 10, 20, 30, 45, 60, 90 and 120 min post pyruvate dosing.

### Mitochondrial isolation

The liver tissue (approximately 100 mg) was finely chopped into a slurry in 1 ml of ice-cold 210 mM mannitol, 70 mM sucrose, 1 mM EDTA, 10 mM HEPES, supplemented with 20 µM 78C (CD38 inhibitor, Sigma) (MIB) buffer. The final pH was adjusted to 7.2 using KOH and freshly supplemented with 0.25% (w/v) fatty acid-free bovine serum albumin (BSA, Roche). The liver slurry was then homogenized by 12 strokes in an ice-cold Teflon Potter Elvehjem homogenizer at 500 rpm. The homogenate was centrifuged at 1,000*g* for 10 min at 4 °C, and the supernatant was further centrifuged for 10 min at 10,000*g* at 4 °C. The mitochondrial pellet was resuspended and washed in MIB without BSA before centrifuging for another 10 min at 10,000*g* at 4 °C. The final mitochondrial pellet was resuspended in MIB without BSA for protein estimation by BCA. The protein concentration was measured by Pierce BCA Protein Assay (Thermo Fisher Scientific). Following isolation and quantification, mitochondria were divided into at least 100 µg replicates in microtubes and centrifuged at 11,800*g* for 10 min to pellet and were used for respiration and NAD^+^ uptake assays or flash-frozen for metabolite analyses, including NAD^+^, NADH and metabolomics.

### NAD^+^ uptake experiments

The isolated mitochondria pellets were resuspended in MiR05 respiration buffer (110 mM mannitol, 0.5 mM EGTA, 3 mM MgCl_2_ 20 mM taurine, 10 mM KH_2_PO_4_, 60 mM K-lactobionate, 20 mM HEPES and 0.1% fatty acid-free BSA, pH adjusted to 7.2 with KOH) containing 5 mM malate and 10 mM pyruvate before being aliquoted into fresh 1.5-ml Eppendorf tubes (typically 200–250 µg mitochondria in a 100-µl volume). The exogenous NAD^+^ at a final concentration of 10 mM was added to the Eppendorf tubes, and the reaction was agitated at 600 rpm in a 37 °C shaker incubator for 0 (handling and washing control) or 30 min. Immediately after the addition of NAD^+^ or after 30 min, 1-ml ice-cold MIB was added to the reaction, and mitochondria were pelleted by centrifugation (11,800*g* for 1 min). The pellet was then washed two more times with ice-cold MIB, spinning 2 min on the final wash, then removing as much buffer as possible. The pellets were either frozen immediately or extracted in 0.6 M perchloric acid.

### NAD^+^ and NADH measurements (enzymatic assay)

The frozen liver tissue (approximately 50 mg) was lysed in 500 µl of ice-cold 0.6 M perchloric acid with a metal bead in the TissueLyser (Qiagen), with a frequency of 20 Hz for 2 min. Tissue lysate was spun at 15,000 x g for 10 mins at 4 °C. The supernatant was diluted to 1:50 in ice-cold 100 mM sodium phosphate buffer (pH 8.0), and NAD^+^ was measured by an enzymatic cycling assay modified from the protocol of Graeff and Lee^40^. In brief, the cycling mix was made fresh immediately before the assay, 5 μl of diluted supernatant or NAD^+^ standard was mixed with 95 µl of cycling mix, which is made with 2% ethanol, 20 μM resazurin, 10 μM flavin mononucleotide, 10 mM nicotinamide, 0.1% BSA in 100 mM sodium phosphate buffer, 100 µg ml^−1^ alcohol dehydrogenase and 10 µg ml^−1^ diaphorase. The NAD^+^ concentration was calculated depending on the rate of resorufin accumulation, which was measured as fluorescence at excitation at 544 nm and emission at 590 nm. The mitochondrial pellets (100–200 µg) were similarly extracted either in 0.6 M perchloric acid or 0.25 M potassium hydroxide in 50% ethanol and were diluted to 1:10 times for NAD^+^ and 1:5 times for NADH in 100 mM sodium phosphate buffer. The same cycling assay was used to measure mitochondrial NAD^+^ and NADH.

NADP was similarly measured from the perchloric acid extract of the liver lysate by an enzymatic cycling assay modified from the protocol of Graeff and Lee^40^ and Zhu et al.^41^. The cycling mix containing 20 μM resazurin, 10 μM flavin mononucleotide, 10 mM nicotinamide, 0.1% BSA in 100 mM sodium phosphate buffer, 11 µg ml^−1^ diaphorase, 1 mM MgCl_2_, 31 mM D-glucose phosphate (G6P), and 0.03 µg ml^−1^ glucose-6-phosphate dehydrogenase from *Leuconostoc mesenteroides* (G6PD, Sigma) was made freshly, and β-NAD phosphate (β-NADP) hydrate was used for the standard curve. Similarly, 0.25 M potassium hydroxide in 50% ethanol extracts of liver lysates were used for NADPH assay following the same protocol described above. The NADP and NADPH concentration was calculated depending on the rate of resorufin accumulation, which was measured as fluorescence at excitation at 544 nm and emission at 590 nm.

### Generation of NAD^+^ sensor

The clonal HeLa and HepG2 ^mito^cpVenus and ^mito^Sensor cell lines were generated by the lentiviral transduction of HeLa cells (ATCC: CCL-2) with virus encoding ^mito^cpVenus or ^mito^Sensor at a MOI (1:1) as referenced in Luongo et al.^11^. All experiments were done in HeLa and HepG2 cells stably expressing either cpVenus or NAD^+^ biosensor in mitochondria.

### Detection of mitochondrial NAD^+^ by Poly-ADP-ribosylation

HEK293 *SLC25A51*-KO cells^42^, stably expressing a mitochondrially targeted construct composed of EGFP and the catalytic domain of PARP1 (mito-EGFP-PARP1cd) were transiently transfected with SLC25A47, SLC25A51, SLC25A52, SLC25A32, *Arabidopsis thaliana* NDT2 (AtNDT2) and plasmid vehicle or left untransfected. HEK293 cells stably expressing the mitochondrial PARP1cd-construct in the context of a functional *SLC25A51* served as further control. The post transfection cell lysates were subjected to immunoblot analysis using anti-PAR, anti-FLAG, anti-GFP and anti-β-tubulin antibodies, with PAR immunoreactivity serving as the readout for mitochondrial NAD^+^ availability. Similarly, HepG2 cells were transiently cotransfected with both mito-EGFP-PARP1cd and selected members of SLC25 protein family (FLAG-tagged human A32, A47 and AtNDT2 and myc-FLAG-tagged human A51, A52 and A47) to detect mitochondrial NAD^+^ availability^43,44^.

### Metabolite extraction (tissue)

The snap-frozen liver pieces (~20 mg) were transferred to 2-ml round-bottom Eppendorf Safe-Lock tubes on dry ice. The samples were then ground into powder with a CryoMill (Retsch) for 1 min at 25 Hz, maintained at a cold temperature using liquid nitrogen. For every 20 mg tissue, 800 μl of −20 °C 40:40:20 (v/v/v) acetonitrile:methanol:water with 0.1 M formic acid was added to the tube, vortexed for 10 s and allowed to sit on ice for 10 min. For every 800 μl of tissue extract, 70 μl of ice-cold 15% (w/v in H_2_O) NH_4_HCO_3_ was added and vortexed to neutralize the sample. The samples were then centrifuged at 21,000*g* for 20 min at 4 °C. The supernatants were then transferred to plastic vials for LC–MS analysis. A procedure blank was generated identically without tissue and was used later to remove the background ions. The mitochondrial pellets (~200 μg) were similarly extracted in 40:40:20 (v/v/v) acetonitrile:methanol:water with 0.1 M formic acid for LC–MS experiments.

### Metabolite measurement by LC–MS

For the untargeted metabolomics, the liquid chromatography separation was achieved by using a Vanquish Horizon UHPLC System (Thermo Scientific) coupled to Q Exactive Plus Mass Spectrometer (Thermo Scientific). A Waters XBridge BEH Amide XP Column (particle size, 2.5 μm; 150 mm (length) × 2.1 mm (internal diameter)) was used for hydrophilic interaction chromatography (HILIC) separation. The column temperature was 25 °C. Mobile phases A of 20 mM ammonium acetate and 22.5 mM ammonium hydroxide in 95:5 (v/v) water:acetonitrile (pH 9.45) and mobile phase B of 100% acetonitrile were used for both ESI-positive and negative modes. The linear gradient eluted from 90% B (0.0–2.0 min), 90% B to 75% B (2.0–3.0 min), 75% B (3.0–7.0 min), 75% B to 70% B (7.0–8.0 min), 70% B (8.0–9.0 min), 70% B to 50% B (9.0–10.0 min), 50% B (10.0–12.0 min), 50% B to 25% B (12.0–13.0 min), 25% B (13.0–14.0 min), 25% B to 0.5% B (14.0–16.0 min), 0.5% B (16.0–20.5 min), 90% B (20.5–25.0 min). The flow rate was 0.15 ml min^−1^. The sample injection volume was 5 μl. Electrospray ionization source parameters were as follows: spray voltage, 3,200 V or −2,800 V, in positive or negative modes, respectively; sheath gas, 35 arbitrary units (arb); aux gas, 10 arb; sweep gas, 0.5 arb; ion transfer tube temperature, 300 °C; vaporizer temperature, 35 °C. LC–MS data acquisition was operated under full-scan polarity-switching mode for all samples. The full scan was set as: orbitrap resolution, 120,000 at m/*z* 200; AGC target, 1 × 10^7^; maximum injection time, 200 ms; scan range, 60–1,000 m/*z*. The raw LC–MS data were converted to mzXML format using the command line ‘msconvert’ utility. The data were analysed via El Maven software. For each sample, the ion count of each metabolite was normalized to the total ion count from all detected metabolites. To determine metabolites that were significantly changed, the log_2_ fold change was calculated from the regenerated versus resected livers, mitochondria and plasma. The significant metabolites were then analysed using an unpaired Student’s *t*-tests and a false discovery rate by MetaboAnalyst 6.0^45^ (listed in the supporting Extended Data Table 1). The volcano plots were generated using Prism 10.0, comparing the −log_2_ fold change to −log_10_ (*P* values). The statistical analysis of metabolite abundance and enrichment pathway was performed using MetaboAnalyst 6.0^45^.

### Mitochondrial respiration assays

The respiration assays on isolated mitochondria were performed in an Oroboros O2k High Resolution Respirometer. For the measurement of complex I-, II- and IV-dependent respiration, 200 μg of liver mitochondria was resuspended into the chamber containing 2 ml of prewarmed MIR05 respiration buffer supplemented with pyruvate (20 mM) and malate (10 mM). After the chamber was sealed, the signal was allowed to stabilize for 5–10 min. ADP (1 mM final concentration) was added to initiate state three respiration. Pericidin (0.5 μM) was added to stop complex I-dependent respiration and followed by succinate (20 mM) to initiate complex II-dependent respiration. Antimycin A (5 μM) was then added to inhibit complex III. A mixture of *N*,*N*,*N*′,*N*′-tetramethyl-*p*-phenylenediamine (TMPD, 0.5 mM) along with ascorbic acid (ASC, 2 mM) was used to measure complex IV respiration, followed by inhibition with sodium azide (5 mM). Complex IV-dependent respiration was quantified by subtracting the azide value from the TMPD value. The fatty acid oxidation was measured by adding malate (10 mM), followed by palmitoyl carnitine (4 mM) and ADP (1 mM). The data were analysed using DatLab software 4.3 (Oroboros Instruments).

### Hepatic TG assay

Hepatic TG was extracted from 30 mg of freeze-clamped liver tissue in 300 μl of cell lysis buffer (150 mM NaCl, 50 mM Tris pH 7.4, 0.1% Triton). The homogenized lysates were diluted to 1:20 in cell lysis buffer, and 10 μl of diluted extract was assayed using the Infinity TG kit (Thermo Fisher Scientific) according to the manufacturer’s protocol.

### ATP determination

The luciferase ATP assay was used to measure ATP from the neutralized perchloric acid extracts of snap-frozen liver tissue (~50 mg) using the ATP Determination Kit (Thermo Fisher Scientific) according to the manufacturer’s protocol. In brief, 5 μl of neutralized extract was diluted (1:100) with 1× ATP buffer, and 10 μl of both samples and standards were assayed using luminescence.

### Immunoblotting

For western blot analysis, the cells were lysed in 20 mM Tris (pH 7.5), 150 mM NaCl, 1% sodium dodecyl sulfate, 1 mM ethylenediaminetetraacetic acid (EDTA) and 1 mM 3-aminobenzamide. Before protein determination using BCA reagent and gel electrophoresis, the lysates were passed ten times through a 23G needle. The cell lysates were probed using anti-PAR 10H (Enzo Life Sciences, ALX-804-220-R100), anti-Flag M2 (Merck/Sigma, F7425), anti-β-tubulin (Abcam, ab179513) and anti-GFP JL-8 (Clontech/TaKaRa, 632381).

Whole liver lysates were probed using anti-acetylated lysine (Cell Signaling Technology, CS9441), anti-Poly/Mono-ADP Ribose (Cell Signaling Technology, CS89190), anti CD38 (R&D systems, AF4947) and HRP-conjugated β-actin (Abcam, ab49900). Mitochondrial proteins were also probed for anti-acetylated lysine (Cell Signaling Technology, CS9441), anti-total OXPHOS (Abcam, ab110413) and anti-VDAC (Abcam, ab14734).

The custom antibodies were made against mouse SLC25A51 protein using the peptide, MMDSEAHEKRPPMLT, in the N-terminal region (homologous to the human SLC25A51 protein). The antibodies were raised in chickens, and anti-sera were used to probe for SLC25A51 expression using mitochondrial pellets isolated from livers overexpressing SLC25A51 and eGFP controls.

The immunoreactive proteins were detected by chemiluminescence using Super Signal West Pico PLUS (Pierce), and the images were captured in a ChemiDoc XRS imaging station (Bio-Rad) using Image Lab 6.01 software.

### RNA isolation and mRNA expression

The total RNA was extracted from frozen liver with Trizol (Sigma-Aldrich). The RNA concentrations were measured using a ThermoFisher Scientific Nanodrop TM spectrophotometer, and 1–2 μg of total RNA was then used to synthesize cDNA using High-Capacity cDNA Reverse Transcription Kit (Applied Biosystems, 4368814) according to the manufacturer’s recommendations. Real-time polymerase chain reaction was performed on an Applied Biosystems 7900HT system with SYBR green master mix (Applied Biosystems). Three technical replicates were obtained for each sample, and a relative standard curve 2^−ΔΔCt^ method was used to quantify the results obtained using gene-specific primers. TATA box binding protein gene and β-actin were used as housekeeping genes. The primers designed in the homologous region of human and mouse *Slc25a51* for quantitative real-time polymerase chain reaction were h/m*SLC25A51* p1 (FW: TACCAACACTTACCAGGCTTTCA, REV: CAAGACATTGCTGAGT-CCATTCC) and procured from Integrated DNA Technologies, Inc.

### Transcriptomics

RNA sequencing and analysis were performed by Novogene. The data were analysed and processed using the cloud platform NovoMagic. In brief, the quantification of gene expression level was performed using featureCounts v1.5.0-p3, and the resulting data were used to calculate fragments per kilobase per million base pairs (FPKM). The differential expression analysis was performed using the DESeq2 R package (1.20.0). The resulting *P* values were adjusted using the Benjamini and Hochberg’s approach for obtaining a false discovery rate (FDR). Genes with an adjusted *P* < 0.05 found by DESeq2 were assigned as differentially expressed.

### Proteomics

The samples were processed and run as previously described^46^. In brief, cryomilled samples were suspended in a solution of 100 mM ammonium bicarbonate and 8 M urea. In total, 50 μg of protein was reduced with dithiothreitol, alkylated with iodoacetamide, digested with trypsin and desalted using in-house StageTips. The peptides were separated using Dionex Ultimate 3000 nanoLC on a C18 column and detected using data-independent acquisition on a Thermo Q Exactive HF. The data were searched using DIA-NN^47^.

The proteomic data were log_2_ normalized and slope-corrected, and missing values were imputed as a distribution around the limit of detection. The significance was calculated by a conditional Mann–Whitney test based on Shapiro–Wilk significance when each comparison group had more than five replicates. In all other comparisons, and in cases where Shapiro–Wilk was not significant, Student’s parametric or non-parametric *t*-test was used on the basis of *F*-test significance^48^.

### Mitochondrial/cytosolic fractionation by NAD-SILEC

HAP1, human haploid cells, were grown in nicotinamide, nicotinic acid and tryptophan-free Dulbecco’s modified Eagle media, custom-made from Corning Life Sciences (PB24024), and were substituted with stable isotope-labelled 1 mg l^−1^ ^13^C_3_,^15^N_1_-nicotinamide (CNLM-9757), 1 mg l^−1^ ^13^C_6_-nicotinic acid (CLM-9954) and 16 mg l^−1^ ^13^C_11_,^15^N_2_-tryptophan (CNLM-2475) from Cambridge Isotope Laboratories. The HAP1 cells were maintained in isotopic labelled media supplemented with dialysed foetal bovine serum for at least eight to nine passages to obtain an efficient >99% NAD-SILEC labelling. The labelling efficiency was tested by isotopologue enrichment analysis with comparison with unlabelled control cells. A total of 20 mg of liver pieces from SLC25A51-OE or eGFP mice was finely chopped into a slurry and immediately transferred in 1 ml of prechilled MIB buffer containing labelled NAD-SILEC HAP1 cells previously grown to confluency in a 10-cm culture dish. Both the liver and cells were homogenized in a Teflon Potter Elvehjem homogenizer at 1,200 rpm for 15 strokes and subjected to fractionation by differential centrifugation^49^. The labelled HAP1 cell and liver tissue controls were homogenized at 1,600 rpm for 16 strokes and 600 rpm for 12 strokes, respectively. For each experiment, 1 μM ^13^C_5_ NAD tracer (CLM-10671) was used to spike whole cell/tissue lysate and cytosolic fractions, as an internal standard for analytical robustness. The whole cell/tissue lysate, high-density debris, cytosol and mitochondrial fractions were processed for analysis by LC–MS with modifications^24,50^. The isotopologue enrichment and fraction labelling were calculated^50^ and plotted in GraphPad Prism 10. Each fraction was run in triplicate for every experiment. The subcellular fractions were subjected to western blot analysis and probed for specific cytosolic proteins (human anti-GAPDHS (MBS 9414022)), anti-GAPDH (Abcam, ab9484), mitochondrial proteins (human anti-mitochondria surface proteins clone 113-1 (Sigma, MAB1273)) and anti-VDAC (Abcam, ab14734) to check the purity of fractions.

### Statistics

Before testing significance, normality was assessed using the Shapiro–Wilk test and equality of variances was tested using Levene’s test^51^. When normality and equal variances were met, a Student’s *t*-test or one- or two-way analysis of variance (ANOVA) was performed when testing two groups, more than two groups with one variable or two independent variables, respectively. When normality was met and there were unequal variances, a Welch’s *t*-test, a Welch’s one-way ANOVA or an adjusted Welch version of the two-way ANOVA using trimmed means was used when testing two groups, more than two groups with one variable or two independent variables, respectively^52^. For non-parametric testing with equal variances, a Mann–Whitney, Kruskal–Wallis, or Scheirer–Ray–Hare^53^ test was performed when testing two groups, more than two groups with one variable or more than one independent variable, respectively. For all cases requiring non-parametric testing with unequal variances, a Kruskal–Wallis test was used. The tests for normality and equality of variances, along with the resulting statistical test selected for each panel, are provided in the statistical source data.

For significant one-way ANOVA or Kruskal–Wallis tests with a single variable, post hoc testing was used to provide individual *P* values for pairwise comparisons by performing Fisher’s least significant difference and Dunn’s test, respectively, not corrected for false discoveries. For experiments involving two independent variables, we report individual *P* values for main effects (for example, genotype and time) and their interaction. Where the interaction term was significant, post hoc comparisons were conducted using Fisher’s least significant difference test, percentile-based bootstrapping methods, and Dunn’s test for two-way ANOVA, two-way Welch ANOVA and Scheirer–Ray–Hare tests, respectively. When the interaction was not significant, no post hoc tests were performed for pairwise comparisons.

A *P* value ≤0.05 was considered significant, with additional thresholds defined in the figure legends. All data points represent biological replicates. The statistical analysis for metabolomics was performed on log_2_ normalized data and analysed by parametric or non-parametric *t*-tests for significance. The statistical tests were performed using R (version 4.4.2)^54^ and GraphPad Prism version 10.5.0 for Windows (GraphPad Software, www.graphpad.com).

### Study approval

All animal work was performed following the guidelines of and with the approval of the University of Pennsylvania’s IACUC (approval protocol number 804892). The work on SLC25A47-knockout mice was approved by IACUC at Beth Israel Deaconess Medical Center.

### Statistical source data

The summary of statistical tests applied to each figure panel and supplementary data output from Shapiro–Wilk and Levene’s tests for each figure panel where applicable can be found in the statistical source data.

### Reporting summary

Further information on research design is available in the Nature Portfolio Reporting Summary linked to this article.

## Supplementary information

Reporting Summary

## Source data

Source Data Fig. 1 Statistical source data.

Source Data Fig. 1 Unprocessed western blots.

Source Data Fig. 2 Statistical source data.

Source Data Fig. 3 Statistical source data.

Source Data Fig. 4 Statistical source data.

Source Data Extended Data Fig. 1 Statistical source data.

Source Data Extended Data Fig. 1 Unprocessed western blots.

Source Data Extended Data Fig. 2 Statistical source data.

Source Data Extended Data Fig. 3 Statistical source data.

Source Data Extended Data Fig. 3 Statistical source data.

Source Data Extended Data Fig. 4 Statistical source data.

Source Data Extended Data Fig. 5 Statistical source data.

Source Data Extended Data Fig. 6 Statistical source data.

Source Data Extended Data Fig. 6 Unprocessed western blots.

Source Data Extended Data Fig. 7 Statistical source data.

Source Data Extended Data Fig. 8 Statistical source data.

Source Data Extended Data Fig. 9 Statistical source data.

Source data Statistical tests performed.

## Figures and Tables

**Extended Data Table 1:**
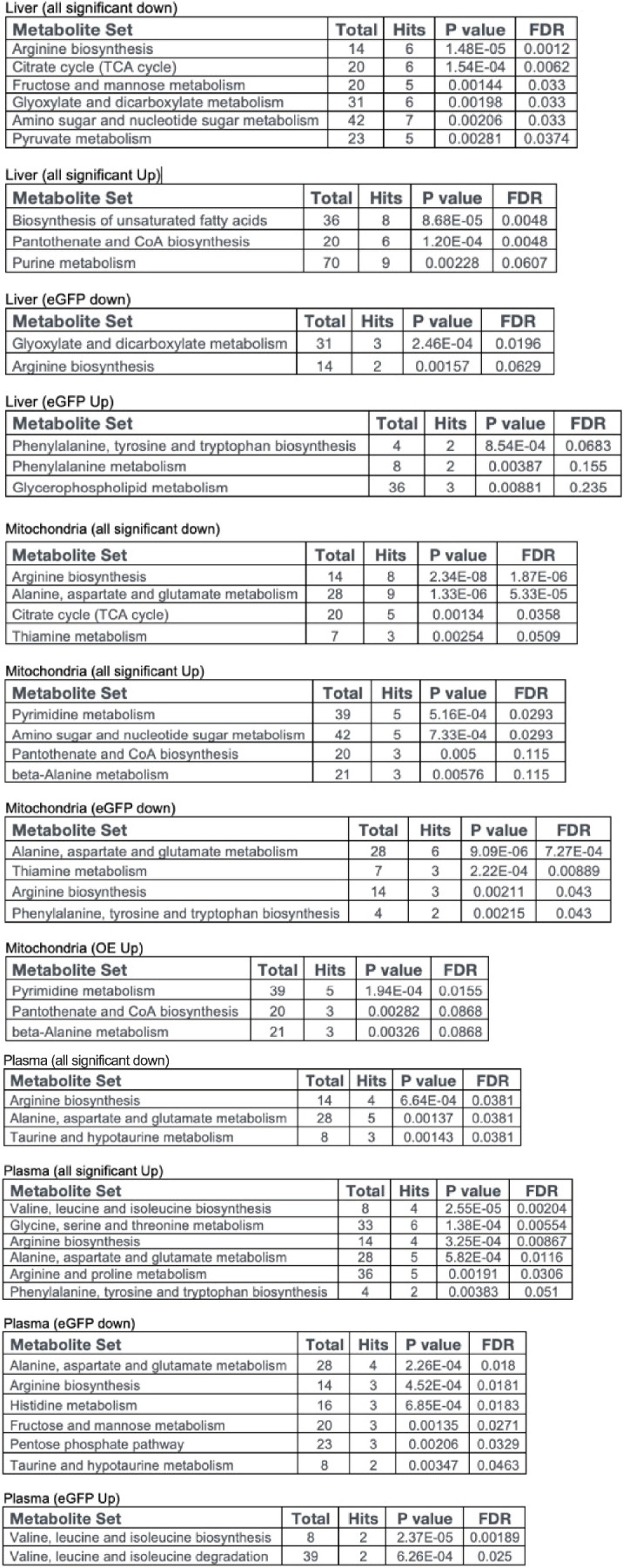
Pathway enrichment analysis during liver regeneration with or without overexpression of SLC25A51

Pathway enrichment analysis output from MetaboAnalyst 6.0^45^ corresponding to Extended Data Fig. 8. Tabulated data representing *P* values from a Student’s t-test and adjusted by a Benjamini–Hochberg correction to obtain false discovery rates (FDR).

## Data Availability

The metabolomics data have been deposited in the MassIVE database under the accession code MSV000098839. The RNA sequencing files have been deposited in the BioProject database under the accession code PRJNA1305469. Proteomics raw files have been deposited in the PRIDE database under the accession code PXD067549. Source data are provided with this paper.
