# Supplementary material for: Hepatocyte mitochondrial NAD+ content is limiting for liver regeneration
Source: Nat Metab. 2025 Nov 20;7(12):2424–37. doi: 10.1038/s42255-025-01408-5 (PMC12727530; doi:10.1038/s42255-025-01408-5)
Supplement: Supplementary file 1 — Reporting Summary [file 42255_2025_1408_MOESM1_ESM.pdf]

nature portfolio

Corresponding author(s): Joseph A Baur

Last updated by author(s): 09/22/2025

Reporting Summary

Nature Portfolio wishes to improve the reproducibility of the work that we publish. This form provides structure for consistency and transparency in reporting. For further information on Nature Portfolio policies, see our Editorial Policies and the Editorial Policy Checklist.

Statistics

For all statistical analyses, confirm that the following items are present in the figure legend, table legend, main text, or Methods section.

n/a	Confirmed	
☐	☒	The exact sample size (*n*) for each experimental group/condition, given as a discrete number and unit of measurement
☐	☒	A statement on whether measurements were taken from distinct samples or whether the same sample was measured repeatedly
☐	☒	The statistical test(s) used AND whether they are one- or two-sided<br>*Only common tests should be described solely by name; describe more complex techniques in the Methods section.*
☒	☐	A description of all covariates tested
☐	☒	A description of any assumptions or corrections, such as tests of normality and adjustment for multiple comparisons
☐	☒	A full description of the statistical parameters including central tendency (e.g. means) or other basic estimates (e.g. regression coefficient) AND variation (e.g. standard deviation) or associated estimates of uncertainty (e.g. confidence intervals)
☐	☒	For null hypothesis testing, the test statistic (e.g. *F*, *t*, *r*) with confidence intervals, effect sizes, degrees of freedom and *P* value noted<br>*Give P values as exact values whenever suitable.*
☒	☐	For Bayesian analysis, information on the choice of priors and Markov chain Monte Carlo settings
☒	☐	For hierarchical and complex designs, identification of the appropriate level for tests and full reporting of outcomes
☐	☒	Estimates of effect sizes (e.g. Cohen's *d*, Pearson's *r*), indicating how they were calculated

Our web collection on statistics for biologists contains articles on many of the points above.

Software and code

Policy information about availability of computer code

Data collection	Histological images were collected using a light microscope (Olympus DP72) coupled with a digital image acquisition system using software cellSens Entry v1.5. QuPath software39 was used to quantify Ki-67 positive cells. Images were captured at 400X magnification using a Nikon Eclipse E600 fluorescence microscope equipped with Qimaging, Q click digital camera. Metabolomics data was obtained from Vanquish 412 Horizon UHPLC System (Thermo Scientific) coupled to Q Exactive Plus Mass Spectrometer (Thermo 413 Scientific). Proteomics data were acquired using Thermo Q Exactive HF and data were searched using DIA-NN. Real-time qPCR data was obtained from ABI QuantStudio v5.0. Immunofluorescence was quantified using QuPath software.
Data analysis	Statistics were performed in GraphPad Prism v10.0 and R (packages factoextra, tidyverse, dyplr, ARTool, rcompanion, WRS2, car; version 4.4.2) Respirometry data analyses were performed in DATLab v7.0. For metabolomics, raw file were converted using "msconvert" in the command line and features were annotated using El Maven v12.1. Proteomics data were searched using DIA-NN (v.2.2.0). RNA-seq analysis was performed by Novogene Inc. Data were analyzed and processed using the cloud platform NovoMagic.

For manuscripts utilizing custom algorithms or software that are central to the research but not yet described in published literature, software must be made available to editors and reviewers. We strongly encourage code deposition in a community repository (e.g. GitHub). See the Nature Portfolio guidelines for submitting code & software for further information.

Data

Policy information about availability of data

All manuscripts must include a data availability statement. This statement should provide the following information, where applicable:

- Accession codes, unique identifiers, or web links for publicly available datasets
- A description of any restrictions on data availability
- For clinical datasets or third party data, please ensure that the statement adheres to our policy

Metabolomics data has been deposited in the MassIVE database under the accession code MSV000098839. RNA-seq files has been deposited in the BioProject database under the accession code PRJNA1305469. Proteomics raw files have been deposited in the PRIDE database under the accession code PXD067549. The authors declare that all source data is available within the study, additional data or inquiries can be directed to the corresponding author.

Research involving human participants, their data, or biological material

Policy information about studies with human participants or human data. See also policy information about sex, gender (identity/presentation), and sexual orientation and race, ethnicity and racism.

Reporting on sex and gender	N/A
Reporting on race, ethnicity, or other socially relevant groupings	N/A
Population characteristics	N/A
Recruitment	N/A
Ethics oversight	N/A

Note that full information on the approval of the study protocol must also be provided in the manuscript.

Field-specific reporting

Please select the one below that is the best fit for your research. If you are not sure, read the appropriate sections before making your selection.

☒ Life sciences ☐ Behavioural & social sciences ☐ Ecological, evolutionary & environmental sciences

For a reference copy of the document with all sections, see nature.com/documents/nr-reporting-summary-flat.pdf

Life sciences study design

All studies must disclose on these points even when the disclosure is negative.

Sample size	Sample sizes were based on the feasibility and animal availability to achieve significant results.
Data exclusions	Data were excluded from animals that exhibited abnormal gait and assessment of pain and dehydration post partial hepatectomy.
Replication	The number of replicates for each experiment is stated in the figure panels and method sections.
Randomization	This study was not randomized as we used the littermates of Slc25a51 heterozygous and wild type mice as controls. For mice overexpressing SLC25A51, we infected with AAV virus expressing human SLC25A51 or with AAV-EGFP as controls. Regardless, we designed experiments in an unbiased way to match age and body weight.
Blinding	Blinding during group allocation was not possible given the availability of the mice. All the partial hepactectomy surgeries were done in a blinded manner. All omics studies were ran in a blinded way.

Reporting for specific materials, systems and methods

We require information from authors about some types of materials, experimental systems and methods used in many studies. Here, indicate whether each material, system or method listed is relevant to your study. If you are not sure if a list item applies to your research, read the appropriate section before selecting a response.

Materials & experimental systems

n/a	Involved in the study
☐	☒ Antibodies
☐	☒ Eukaryotic cell lines
☒	☐ Palaeontology and archaeology
☐	☒ Animals and other organisms
☒	☐ Clinical data
☒	☐ Dual use research of concern
☒	☐ Plants

Methods

n/a	Involved in the study
☒	☐ ChIP-seq
☒	☐ Flow cytometry
☒	☐ MRI-based neuroimaging

Antibodies

Antibodies used	Anti-poly-ADP-ribose 10H (Enzo Life Sciences, ALX-804–220-R100), anti-Flag M2 (Merck/Sigma, 467 F7425), anti-β-tubulin (Abcam, ab179513), anti-GFP JL-8 (Clontech/TaKaRa , 632381), anti-acetylated lysine (Cell Signaling, CS9441), anti-Poly/Mono-ADP Ribose (Cell Signaling, CS89190), anti-CD38 (R&D systems, AF4947), HRP-conjugated β-actin (Abcam, ab49900), anti-total OXPHOS (Abcam, ab110413), anti-VDAC (Abcam, ab14734), and anti Ki67(Abcam, ab16667). Custom antibodies were made against mouse SLC25A51 protein using the peptide, MMDSEAHEKRPPMLT, in the N-terminal region (homologous to the human SLC25A51 protein).
Validation	All commercial antibodies were validated by the manufacturers and have been cited in publications.

Eukaryotic cell lines

Policy information about cell lines and Sex and Gender in Research

Cell line source(s)	HEK293, HeLa, and HepG2 were purchased from ATCC.
Authentication	All cell lines were authenticated by STR profiling as per ATCC.
Mycoplasma contamination	All cell lines were routinely tested negative from mycoplasma.
Commonly misidentified lines (See ICLAC register)	N/A

Animals and other research organisms

Policy information about studies involving animals; ARRIVE guidelines recommended for reporting animal research, and Sex and Gender in Research

Laboratory animals	Animals were housed in groups of four to five mice per cage in a pathogen-free barrier facility kept at ambient temperature in a 12-hour light-dark cycle with free access to food and water. All the mouse strains used in this study are in C57BL/6NJ background. The male mice used for partial hepatectomy experiments were 10-16 weeks old.
Wild animals	No wild animals were used in this study.
Reporting on sex	Only males were used in the study.
Field-collected samples	No field-collected samples were used in this study.
Ethics oversight	All animal work was performed following the guidelines of and with the approval of the University of Pennsylvania's IACUC (Approval Protocol number 804892). Work on SLC25A47 KO mice was approved by IACUC at Beth Israel Deaconess Medical Center.

Note that full information on the approval of the study protocol must also be provided in the manuscript.

Plants

Seed stocks	N/A
Novel plant genotypes	N/A
Authentication	N/A
