# Supplementary material for: Hepatocyte mitochondrial NAD+ content is limiting for liver regeneration
Source: Nat Metab. 2025 Nov 20;7(12):2424–37. doi: 10.1038/s42255-025-01408-5 (PMC12727530; doi:10.1038/s42255-025-01408-5)

Fig. 1m- n Immunoblots showing PAR immunoreactive proteins as readouts for mitochondrial NAD^+ availability in HEK293 (m) and HepG2 (n) cells expressing mitoPARP1cd and the indicated mitochondrial carrier proteins

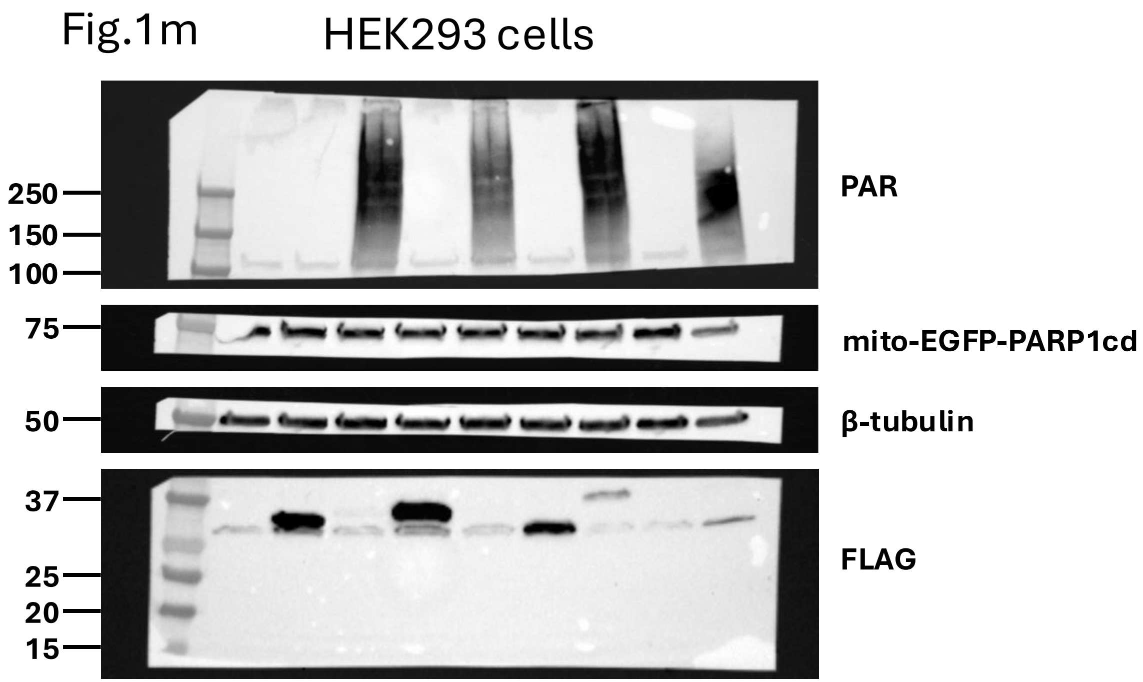

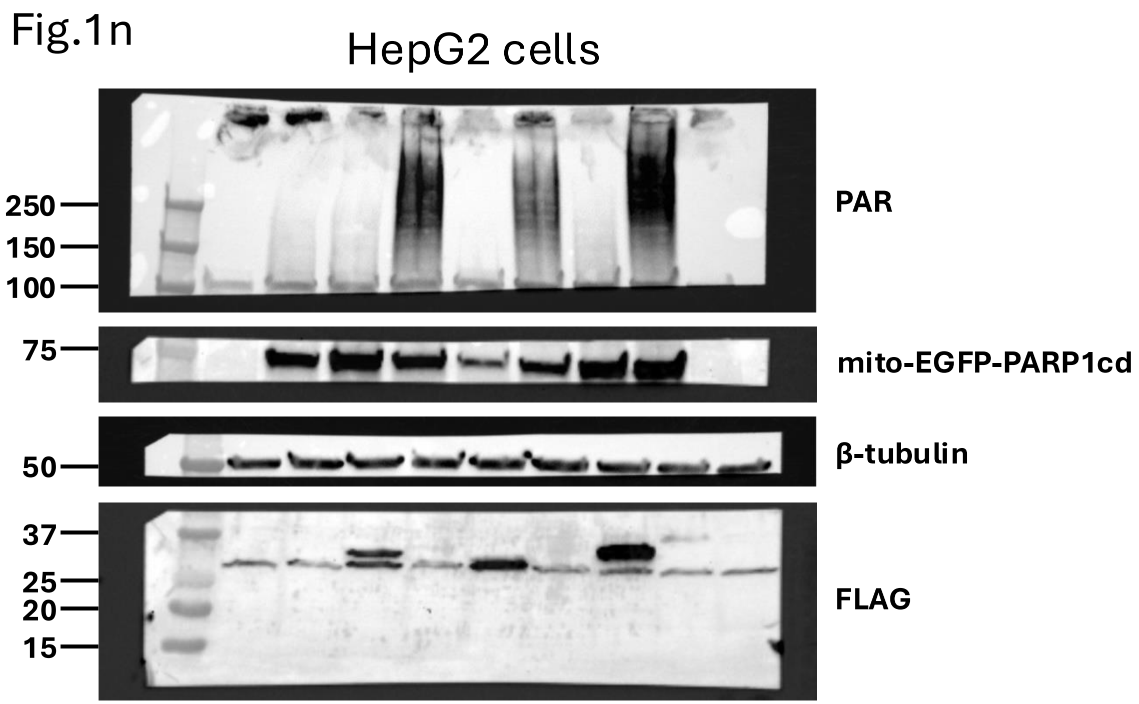

Supplement: Supplementary file 3 — Unprocessed western blots. [file 42255_2025_1408_MOESM3_ESM.pdf]
