# Supplementary material for: Hepatocyte mitochondrial NAD+ content is limiting for liver regeneration
Source: Nat Metab. 2025 Nov 20;7(12):2424–37. doi: 10.1038/s42255-025-01408-5 (PMC12727530; doi:10.1038/s42255-025-01408-5)

Extended data Fig. 1f Western blot showing enrichment of Cox IV (mitochondrial) and depletion of GAPDH (cytosolic) in mitochondrial fractions isolated from HeLa cells

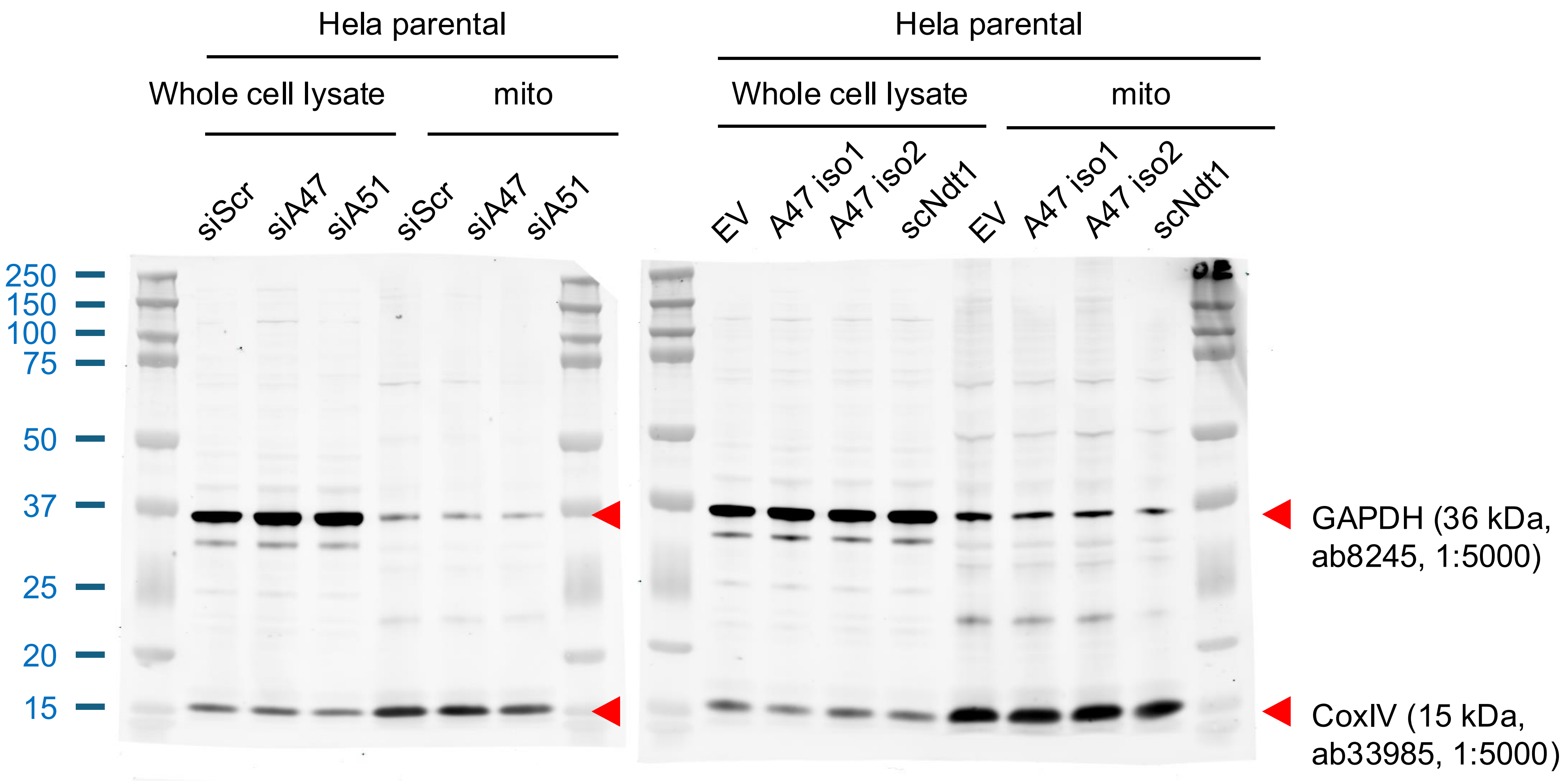

Extended data Fig. 1i Western blot showing overexpression of SLC25A51 and SLC25A47 in HepG2 cells.

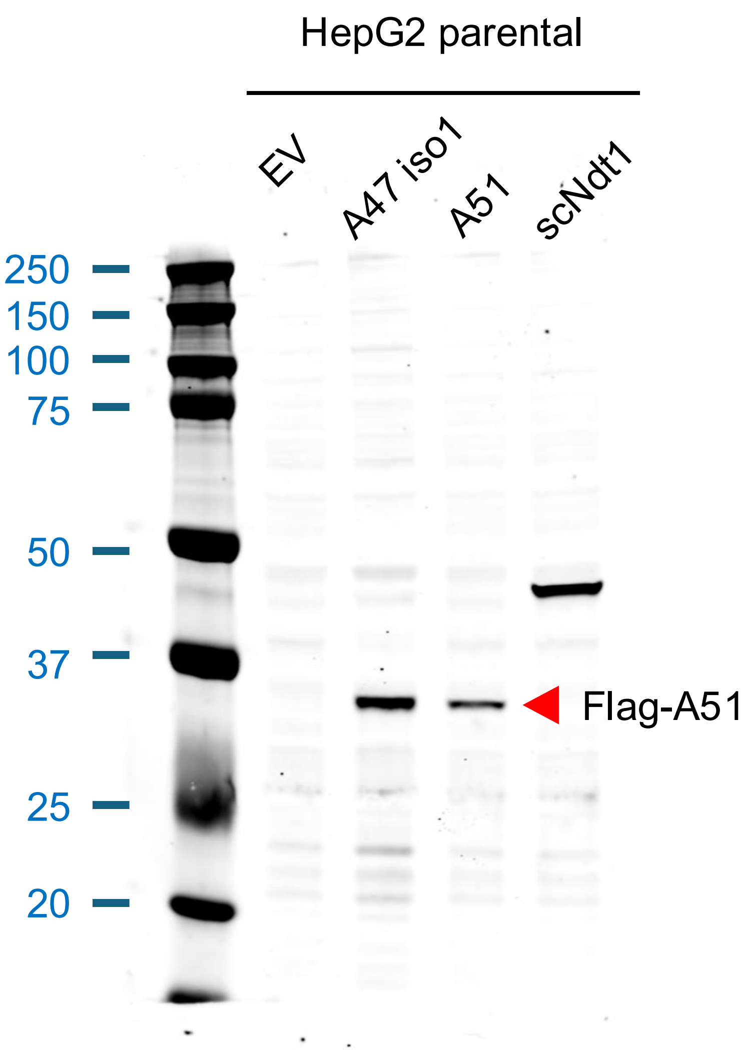

Flag antibody (F1804)
1:3000

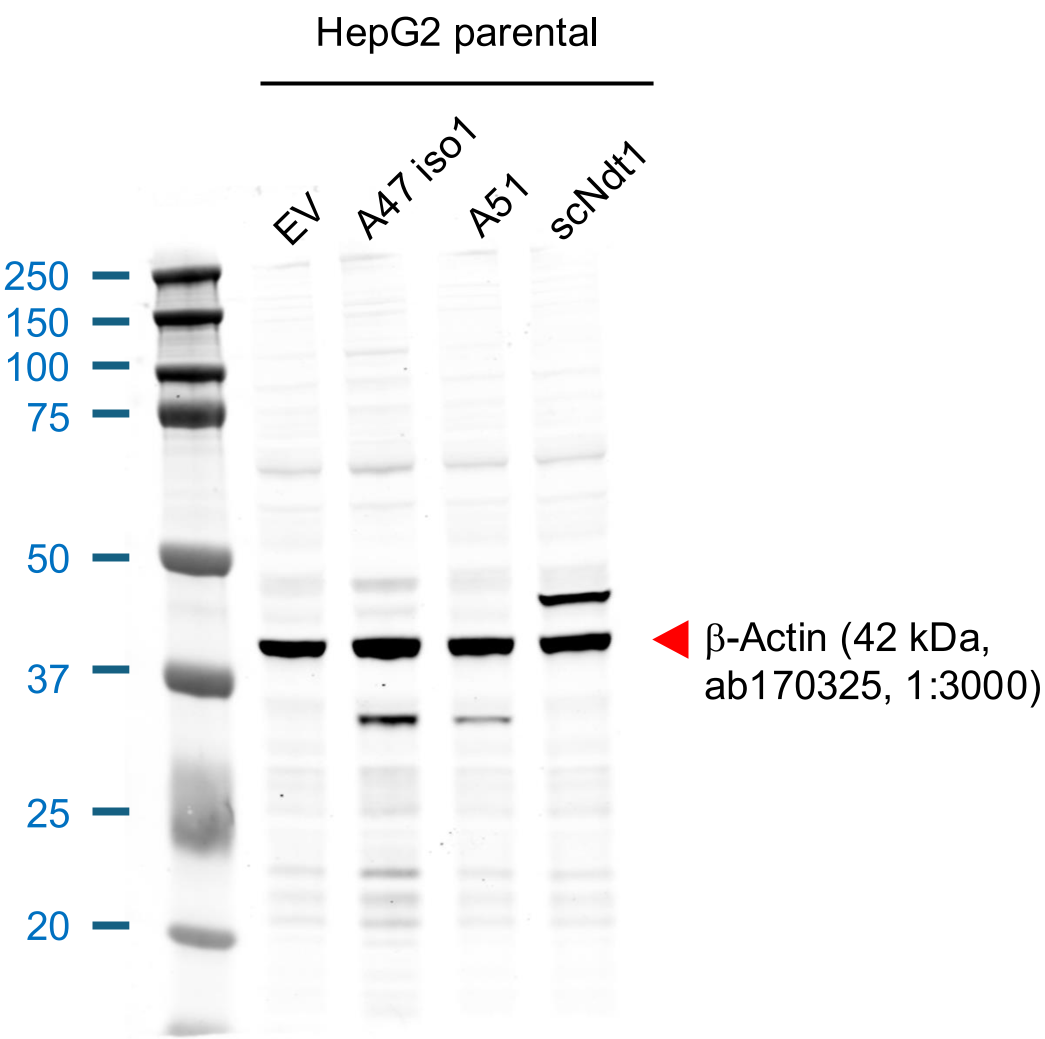

Supplement: Supplementary file 8 — Unprocessed western blots. [file 42255_2025_1408_MOESM8_ESM.pdf]
