# Supplementary material for: Hepatocyte mitochondrial NAD+ content is limiting for liver regeneration
Source: Nat Metab. 2025 Nov 20;7(12):2424–37. doi: 10.1038/s42255-025-01408-5 (PMC12727530; doi:10.1038/s42255-025-01408-5)

Extended data Fig. 3i Immunoblot from liver and isolated mitochondria from pre and 48h post PHx probed for SLC25A51 protein

Isolated liver mitochondria pre-PHx probed chicken anti sera for SLC25A51 (#UP-C120)

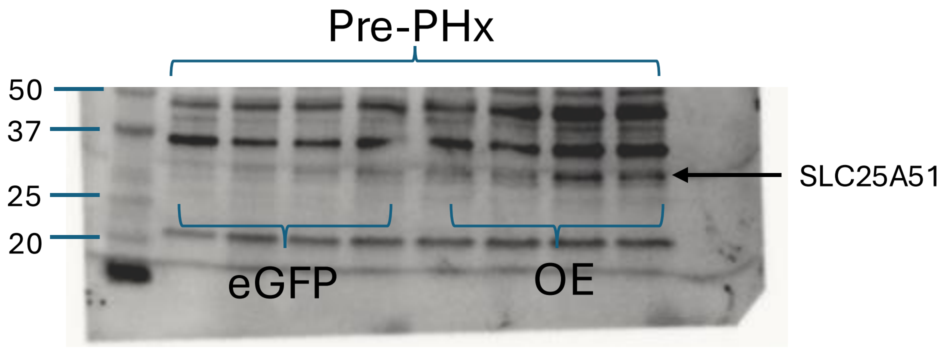

Isolated liver mitochondria post-PHx probed chicken anti sera for SLC25A51 (#UP-C120)

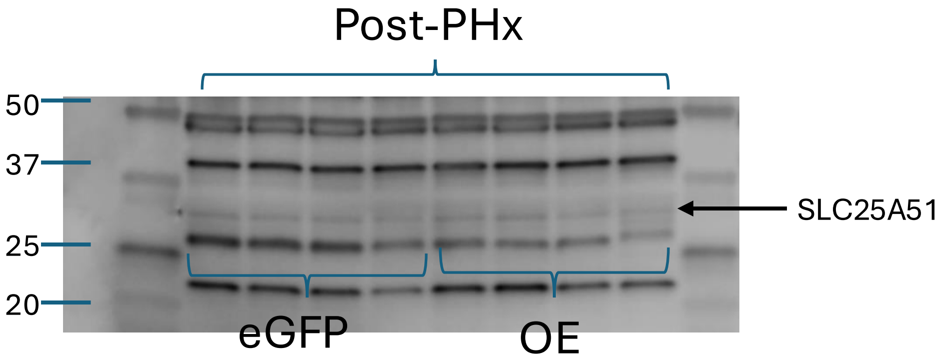

Liver mitochondria pre-PHx-VDAC(ab14734)

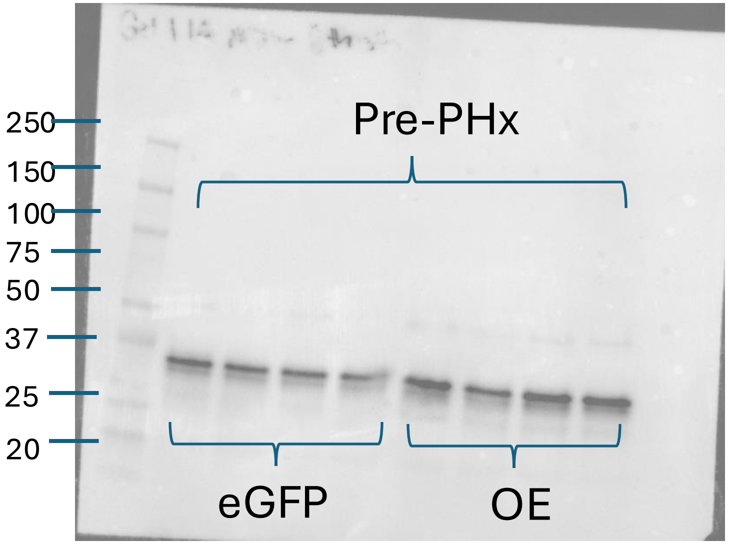

Liver mitochondria post-PHx-VDAC(ab14734)

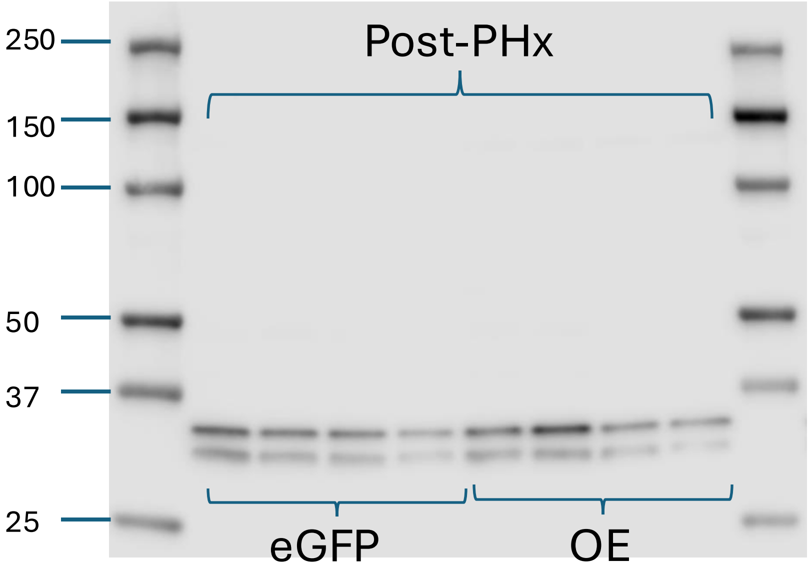

Extended data Fig. 3p Validation of subcellular fractionation by immunoblots

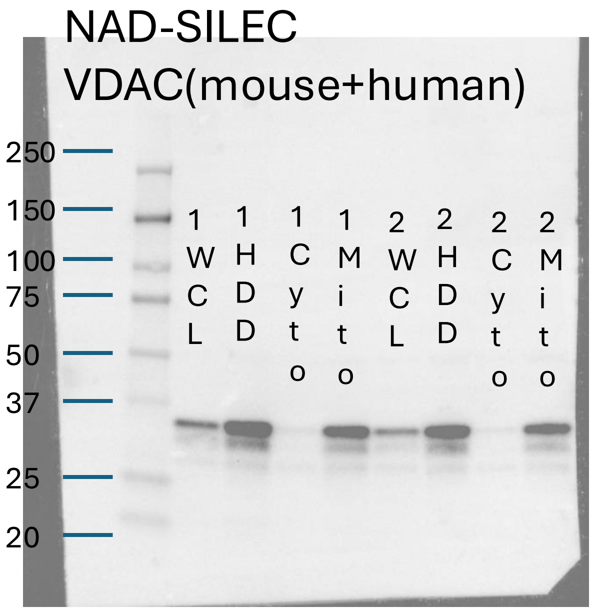

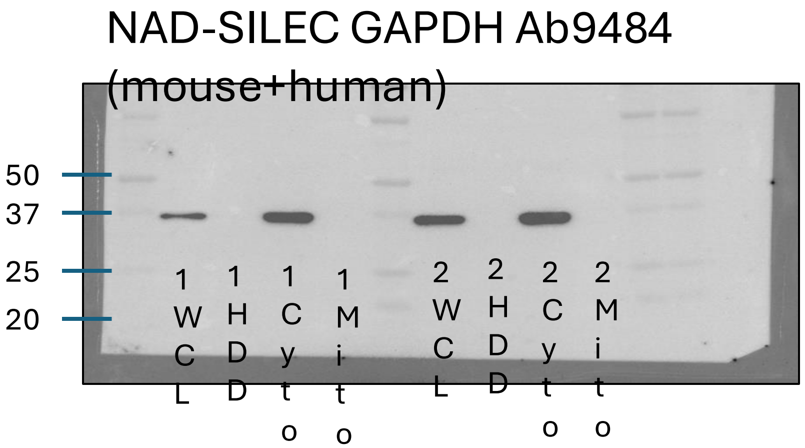

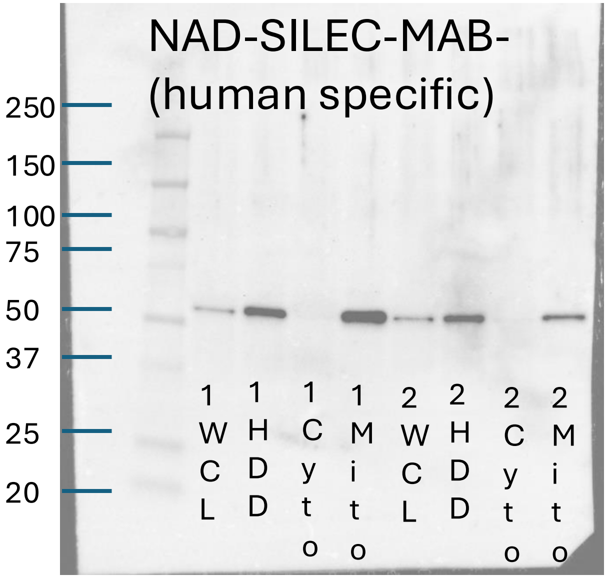

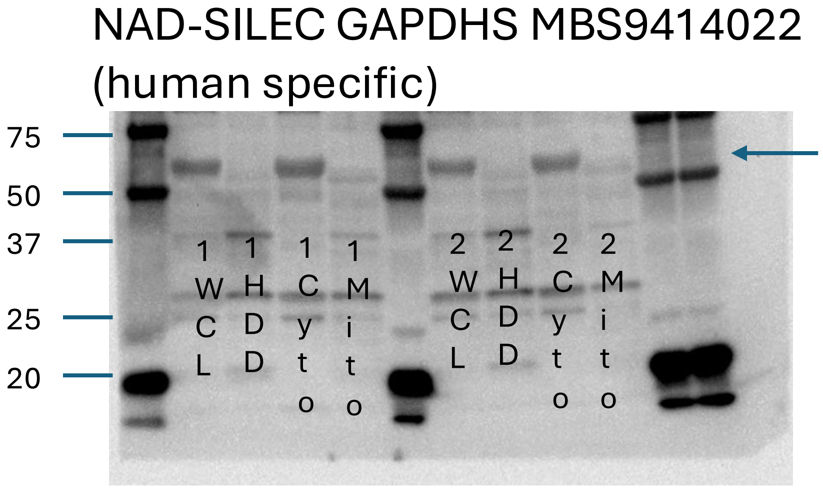

Supplement: Supplementary file 11 — Statistical source data. [file 42255_2025_1408_MOESM11_ESM.pdf]
