# Supplementary material for: Hepatocyte mitochondrial NAD+ content is limiting for liver regeneration
Source: Nat Metab. 2025 Nov 20;7(12):2424–37. doi: 10.1038/s42255-025-01408-5 (PMC12727530; doi:10.1038/s42255-025-01408-5)

Extended data Fig.6a Whole liver tissue lysates probed for acetylated lysine (Ac-K)

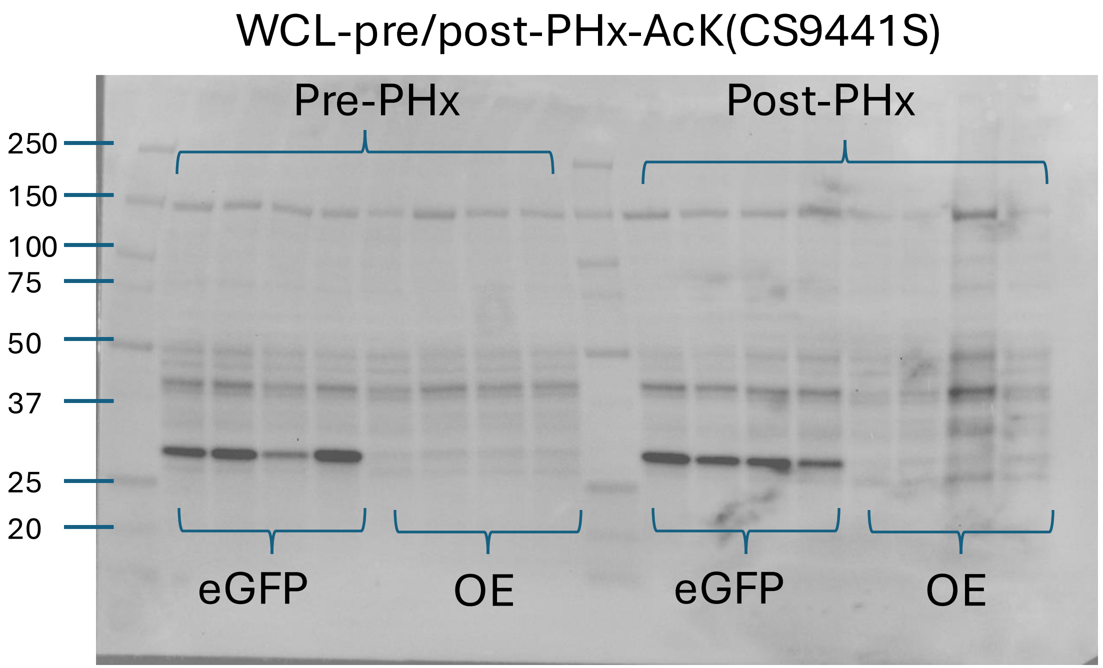

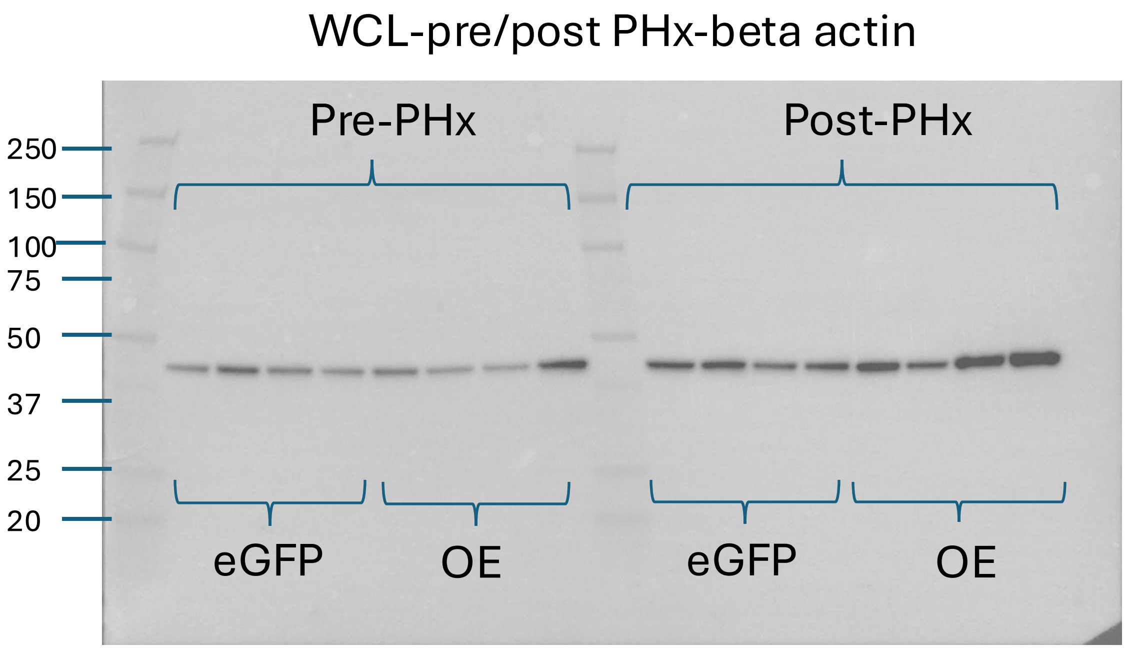

Extended data Fig.6b Isolated liver mitochondria probed for acetylated lysine (Ac-K)

Mitochondria pre/post PHx-Ac K(CS9441S)

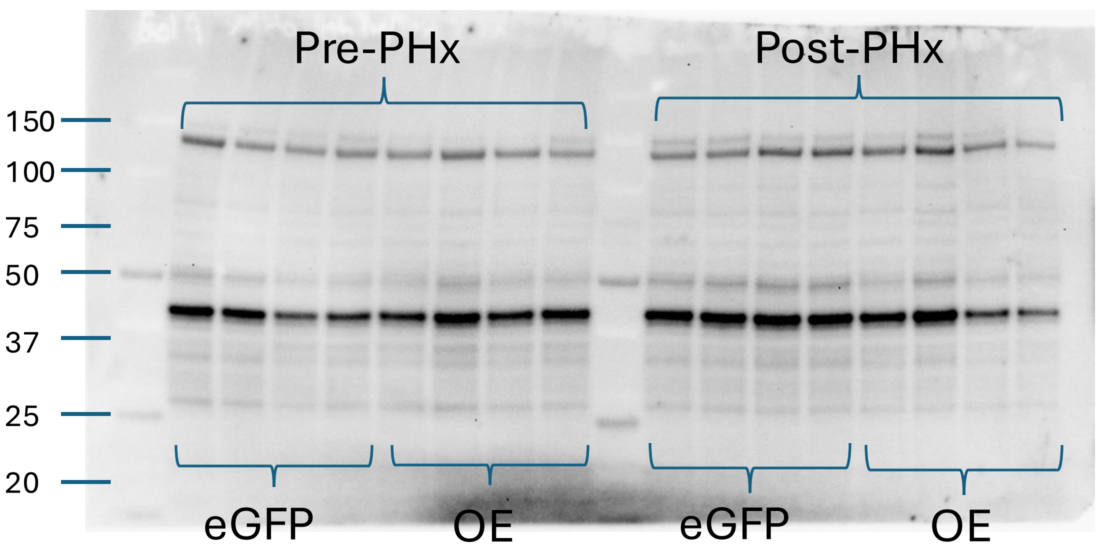

Mitochondria pre/post PHx-VDAC(ab14734)

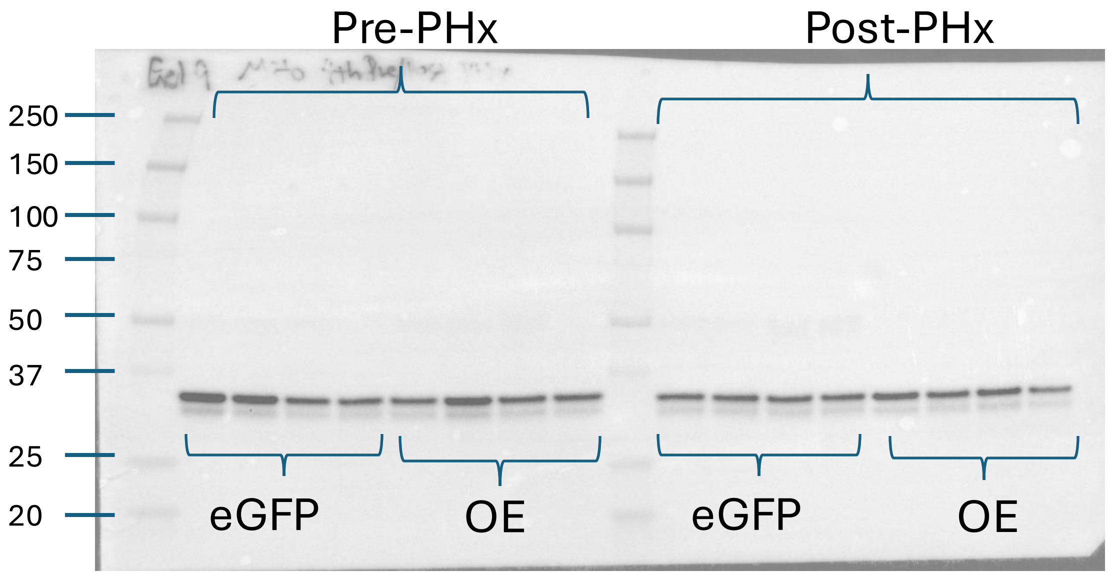

Extended data Fig.6c Total PARylation blot from whole tissue lysates

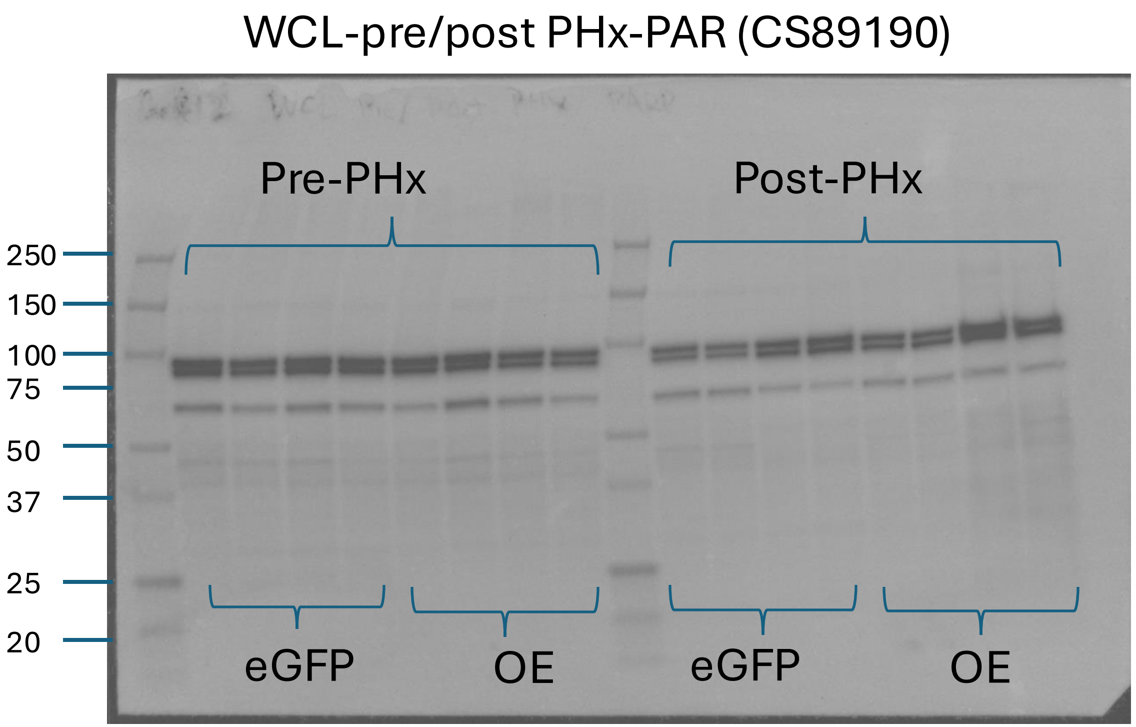

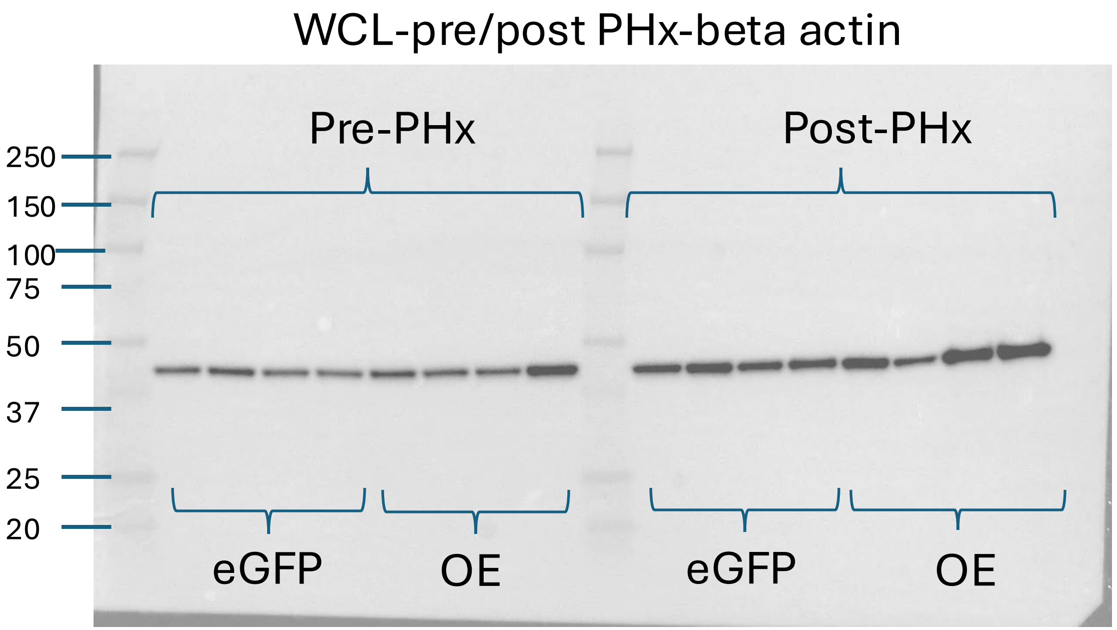

Extended data Fig.6e Liver tissue lysates probed for CD38

WCL-pre/post PHx-CD38 (AF4947)

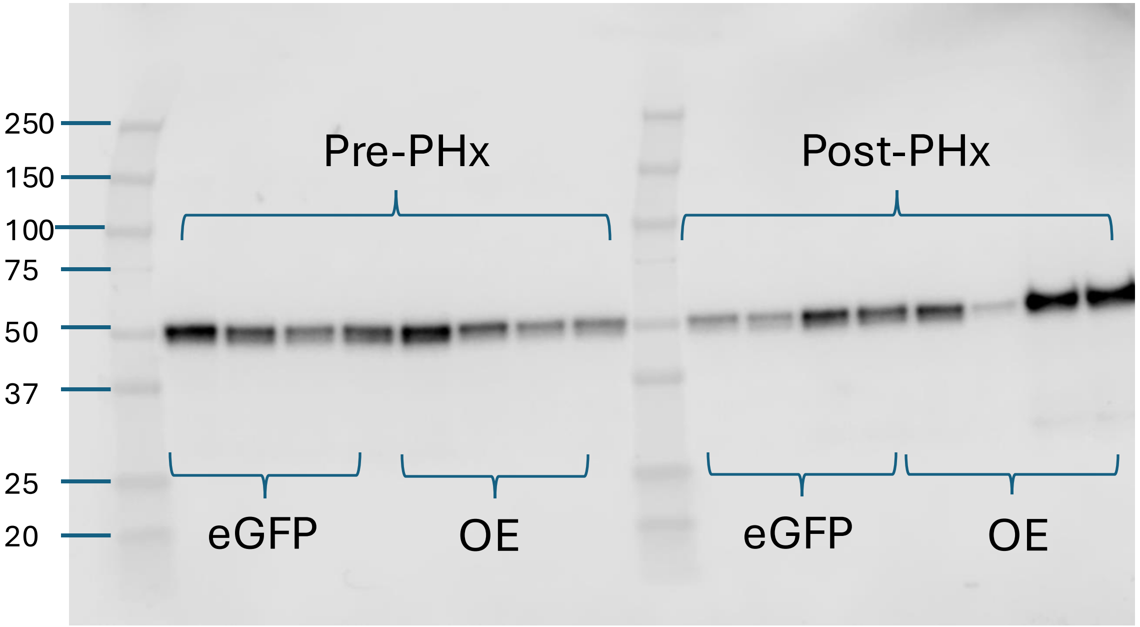

WCL-pre/post PHx-beta actin

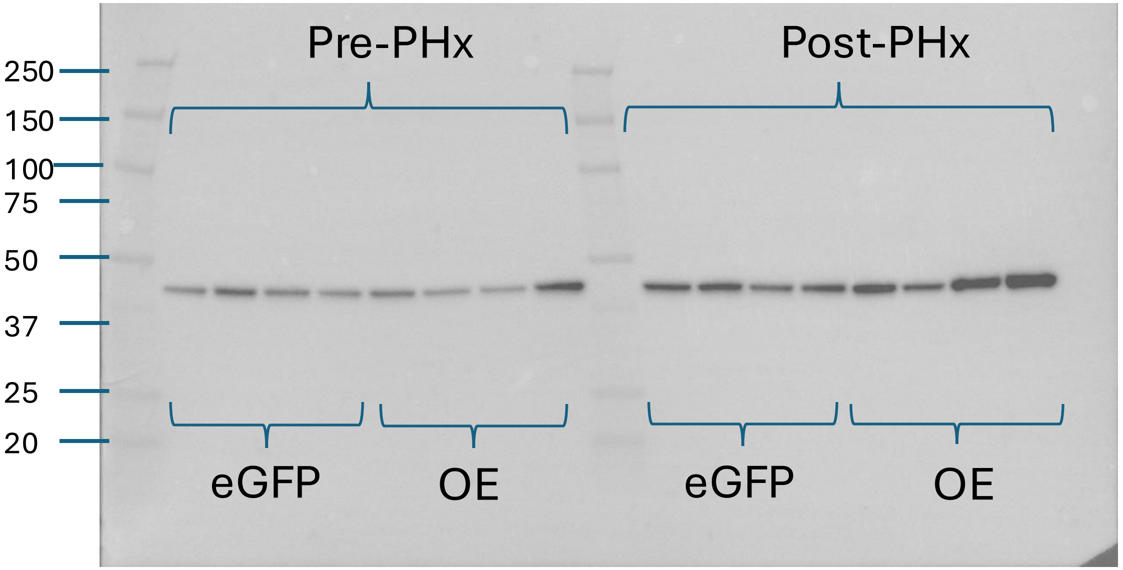

Extended data Fig. 6g Protein complexes in the electron transport chain from isolated mitochondria

Liver mitochondria- pre PHx -OXPHOS

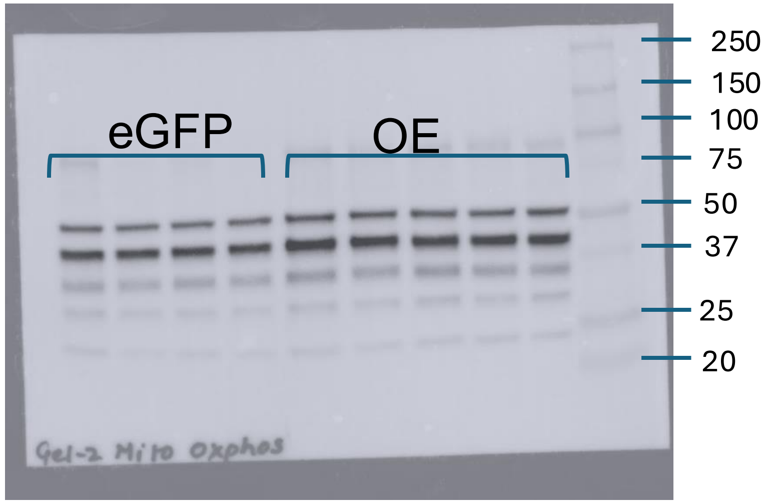

Liver mitochondria- pre PHx -VDAC

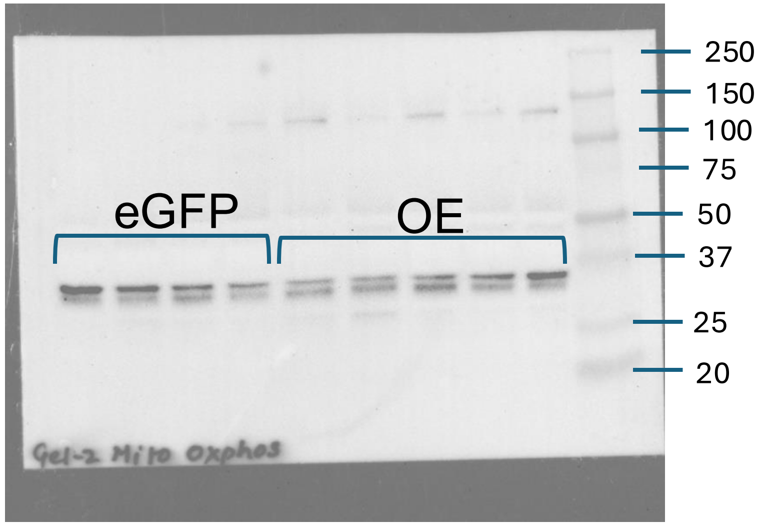

Supplement: Supplementary file 15 — Unprocessed western blots. [file 42255_2025_1408_MOESM15_ESM.pdf]
